# Spatial-temporal characteristics and causes of changes to the county-level administrative toponyms cultural landscape in the eastern plains of China

**DOI:** 10.1371/journal.pone.0217381

**Published:** 2019-05-28

**Authors:** Yingying Wang, Yingjie Wang, Lei Fang, Shengrui Zhang, Tongyan Zhang, Daichao Li, Dazhuan Ge

**Affiliations:** 1 State Key Laboratory of Resources and Environmental Information System, Institute of Geographic Sciences and Natural Resources Research, CAS, Beijing, China; 2 University of Chinese Academy of Sciences, Beijing, China; 3 Spatial Information Research Center of Fujian, Fuzhou University, Fuzhou, China; 4 College of Geography, Nanjing Normal University, Nanjing, China; National Taiwan University, TAIWAN

## Abstract

As part of the cultural landscape, administrative toponyms do not only reflect natural and sociocultural phenomena, but also help with related management and naming work. Historically, county-level administrative districts have been stable and basic administrative regions in China, playing a role in the country’s management. We explore the spatio-temporal evolutionary characteristics of the county-level administrative toponyms cultural landscape in China’s eastern plains areas. A Geographical Information System (GIS) analysis, Geo-Informatic Tupu, Kernel Density Estimation, and correlation coefficients were conducted. We constructed a GIS database of county-level administrative toponyms from the Sui dynasty onward using the Northeast China, North China, and Yangtze Plains as examples. We then summarized the spatio-temporal evolutionary characteristics of the county-level administrative toponyms cultural landscape in China’s eastern plains areas. The results indicate that (1) the number of toponyms has roughly increased over time; (2) toponym densities on the three plains are higher than the national average in the corresponding timeframe since the Sui; and (3) county-level administrative toponyms related to mountains and hydrological features accounted for more than 30% of the total in 2010. However, the percentage of county-level administrative toponyms related to natural factors on the three plains has decreased since the Sui. To explore the factors influencing this spatio-temporal evolution, we analyzed the correlations between the toponyms and natural factors and human/social factors. The correlation degree between toponym density and population density is the highest, and that between toponym density and Digital Elevation Model (DEM) the lowest. Temperature changes were important in toponym changes, and population changes have influenced toponym changes over the last 400 years in China.

## Introduction

A place begins to exist when people assign it a name and meaning, differentiating it from the larger, undifferentiated space [1]. Thus, toponyms (or place names) are names given to places by people. Toponyms are also defined as names for natural and manmade geographic features assigned to particular spaces [2]. As a cultural landscape, toponyms are the preservation and mark of regional culture on the surface level, and directly reveal the spatial distribution of languages and ethnic origins. The term “toponyms cultural landscape” refers to the group characteristics of toponyms formed by consistent factors in a certain area [3]. Moreover, the toponyms cultural landscape does not only reflect the characteristics of the historical and current natural environment, but also records information such as major political changes, the prosperity or decline of a nation, ethnic migration, religious beliefs, and military activities [4, 5]. Toponyms are frequently used to label, identify, and locate sites in space, and are subjective explanations by local inhabitants from the time of naming [6]. In China, studies concerning toponyms at the earliest stage appeared alongside academic works on China’s ancient geography, such as *Shang Shu·Yu Gong*, *Classic of Mountains and Seas* (*Shan Hai Jing*), *Geographical Records on the History of the Han Dynasty*, and *Commentary on the Water Classic* (*Shui Jing Zhu*), which all recorded important information on toponyms [7]. Because of their increasing significance, studies on toponyms attracted the attention of the government and academia. During the Republic of China (ROC) (1912–1949), *The Great Dictionary of Chinese Toponyms* and *Historical Records Test Names* were representative works of Chinese toponymy. Following the founding of the People's Republic of China (PRC) in 1949, the periodical *China Toponyms* was published in 1984, and featured many scholars’ academic achievements regarding toponyms [7, 8]. After the 1^st^ Toponyms Census of China in the 1980s, many works were published that interpreted toponyms from the perspectives of linguistics, historiography, culturology, and geography [9–15]. That established the theoretical foundation for the study of toponyms.

Toponymy developed in the occidental countries in the late 19^th^ century as an independent discipline. For example, the United States established the *Board on Geographic Names* in 1890 [16]; Henry Gannett introduced the origin of toponyms of the United States in 1905 [17]; George Rippey Stewart published the book entitled *Names on the land*: *a historical account of placenaming in USA* in 1982 [18]. In the 21^st^ century, modern research on toponyms tends to focus on quantitative and visualized analyses. For example, the Geographic Information System (GIS) is widely applied in the study of geographical names, and a research method combining cartography with geo-visualization techniques is playing an important role in the quantification and visualization of toponyms. In general, research has focused on exploring the correlation between toponyms and landscape changes [19]; using toponyms to interpret historical land use changes or reconstruct past land use [6, 20]; constructing GIS databases of ancient and modern toponyms [21]; utilizing toponyms to indicate recent climate changes [22]; analyzing the spatial distribution characteristics of toponyms based on a GIS approach, including dialectic toponyms, rural toponyms, and toponyms in ethnic minority languages [23–26]; and using GIS technology to study the cultural landscape of toponyms [3, 27–29].

Although scholars worldwide have investigated toponym features, few have paid attention to the characteristics and intrinsic mechanisms of the evolution of the toponyms cultural landscape at different spatial and temporal scales, and few researches were visualized using maps to intuitively and accurately clarify this evolution. Thus, this study focuses on the changes to the county-level administrative toponyms cultural landscape in China’s eastern plains areas to explore their influencing factors. This study provides theoretical and practical operational guidance for rationally optimizing administrative divisions and toponym management, which could help protect the cultural heritage of toponyms in China.

## Data and methods

### Case areas

As linguistic fossils, toponyms have a mark function for the changes of natural environment and social environment in different historical periods. Administrative toponyms are an integral part of the toponyms cultural landscape, and an important tool for state administration. Further research on administrative toponyms can reveal natural and sociocultural phenomena, and be used for managing and naming places.

County-level administrative districts are the most stable and basic administrative regions in China’s history, and have played a key role in the management of the country. As the generic part of administrative toponyms, “county” dates to China’s Spring and Autumn Period (770–476 BC). Since the nationwide implementation of the prefecture and county system during the Qin dynasty (221–207 BC), “county” became widespread throughout the country [14].

**Fig 1:**
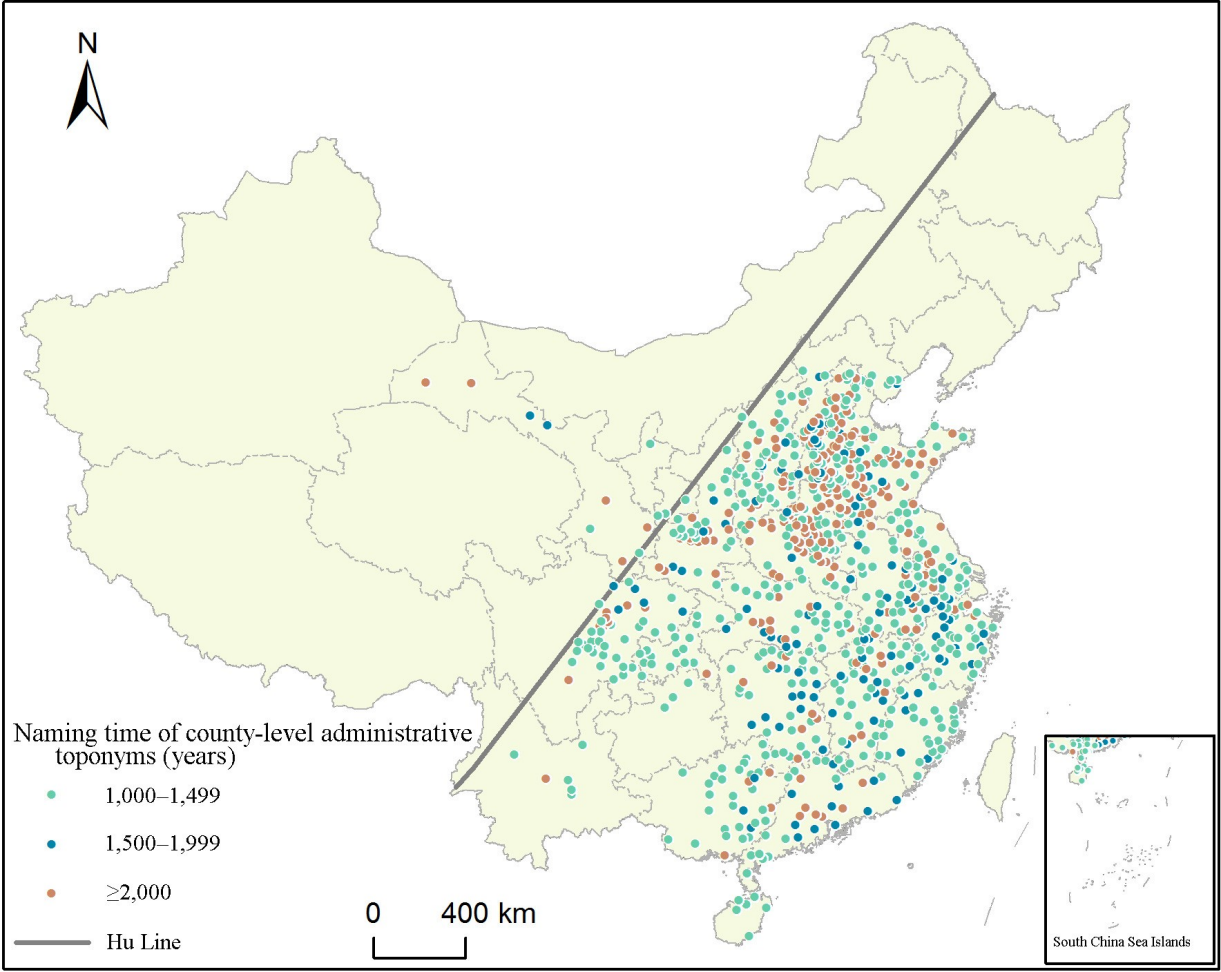
Distribution map of county-level administrative toponyms with the time of being named from 1,000 years ago. The dots represent data on county-level administrative toponyms with naming time from 1,000 years ago. The green dots represent county-level administrative toponyms being named for 1,000–1,499 years, blue dots those being named for 1,500–1,999 years, and red dots those being named for 2,000 years ago.

According to preliminary statistics from the *Dictionary of Chinese Historical Toponyms* [30] and *The New Century Chinese City View* [31], less than 3% of county-level administrative regions still use their names from the Qin and Han dynasties. As shown in Fig 1, 795 county-level administrative toponyms are more than 1,000 years old, of which 331 are more than 1,500 years old and 215 older than 2,000 years. Furthermore, the Hu Line divides China into an eastern and western part. Nearly 98% of county-level administrative toponyms are located in the eastern part, with a concentrated distribution in the plains areas. The dots and polygons data of administrative divisions of China at a scale of 1:1,000,000 (Fig 1) were obtained from National Catalogue Service For Geographic Information [32].

**Fig 2:**
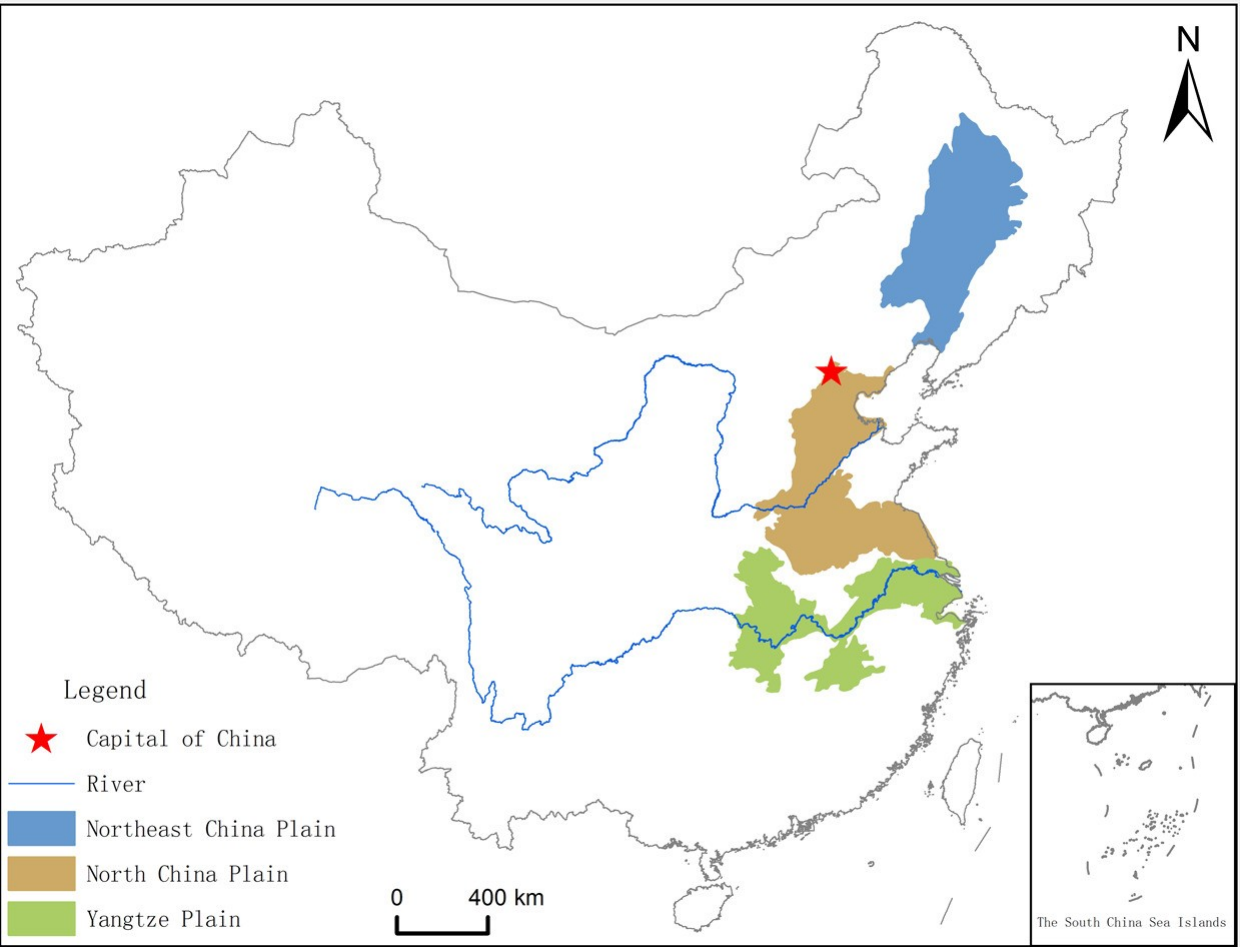
Map of the case study areas.

In addition, the plains areas have the most intimate human-land relationship, and because humans have recognized and reconstructed the natural environment, administrative toponyms in these areas account for a sizable proportion of the total. Furthermore, we employed county-level administrative toponyms located in the Northeast China Plain, North China Plain, and Yangtze Plain (Fig 2) as our study object for the following reasons. First, the Northeast China Plain, North China Plain, and Yangtze Plain are three great plains located in the eastern part of China, and have relatively high representativeness. Second, we selected these three plains as the case study areas because of data availability and processing workload. Third, considering that toponyms are in the category of linguistics, we selected the Northeast China Plain to analyze the influence of minority languages on toponyms to compare it with the North China Plain and Yangtze Plain, even though the Northeast China Plain developed later. The polygons data of China (Fig 2) were obtained from National Catalogue Service For Geographic Information [32].

### Data sources and preparation

For the purposes of this study, the historical toponyms data were acquired from *The Historical Atlas of China* [33]. We vectorized the paper version of the atlas. The dots and polygons data of administrative divisions of China at a scale of 1:1,000,000 were obtained from National Catalogue Service For Geographic Information [32]. Moreover, to explore the influencing factors of the spatio-temporal evolutionary characteristics of these toponyms, we collected GDP data in 2010 at the county level from the RESDC [34]and population data at the county level from the 6^th^ Census of China (2010) [35]. In addition, DEM data at a resolution of 30 m and grid data of China’s population and GDP in 2010 (at a 1-km gridded resolution) were provided by the RESDC [34]. These grid data were used to calculate the correlation coefficients, as explained in the following sections. For historical population data, we referred to statistical data from the National Earth System Science Data Sharing Infrastructure, National Science & Technology Infrastructure of China [36]. Other data were sorted from the *Dictionary of Chinese Historical Toponyms* [30], *The New Century Chinese City View* [31], *The Changes of Administrative Divisions in Chinese History* [37], *Encyclopedic Dictionary of Ancient and Modern Chinese Geographical Names* [38] and *China's Toponyms Anecdotes Dictionary* [39].

### Research methods

#### Kernel density estimation method

The Kernel Density Estimation (KDE) method uses a moving cell (equivalent to a window) to estimate the density of a point or line pattern. It is generally defined as *x*_*1*_, *x*_*2*_…, where *x*_*n*_ are independent and identically distributed samples extracted from the distribution density function f, and f(*x*) is the value estimated at given point *x*. Usually, researchers adopt the Rosenblatt-Parzen kernel estimation [40].

$$f\left(x\right)=\frac{1}{nh}\sum_{i=1}^{n}\left\{k\left[\frac{d\left(x,x_{i}\right)}{h}\right]\right\}$$ (1)

In this expression, *n* denotes the number of toponyms in the range scale, while *k*(·) is the kernel density function of the toponyms, *h* the distance threshold, and *d(x*, *x*_*i*_*)* the Euclidean distance between the estimated point *x* and sample *x*_*i*_.

The KDE method uses the distance attenuation bandwidth to represent an object's spatial influence domain, which is suitable for situations that consider the influence of regions [41]. Therefore, we used the KDE method to study changes in the toponym density of the administrative toponyms cultural landscape in China’s eastern plains areas since the Sui dynasty.

#### Correlation coefficients

We selected data on DEM, GDP, and the population spatial distribution (at a 1-km gridded resolution) in 2010 as experimental data to calculate the correlation coefficients between toponym density and natural, economic, and demographic factors.

$$R_{\mathrm{DEM}}=\frac{\sum_{i=1}^{n}\left(x_{i}‐\bar{x}\right)\left(a_{i}-\bar{a}\right)}{\sqrt{\sum_{i=1}^{n}{\left(x_{i}‐\bar{x}\right)}^{2}\sum_{i=1}^{n}{\left(a_{i}-\bar{a}\right)}^{2}}}$$ (2)

$$R_{\mathrm{GDP}}=\frac{\sum_{i=1}^{n}\left(x_{i}‐\bar{x}\right)\left(b_{i}-\bar{b}\right)}{\sqrt{\sum_{i=1}^{n}{\left(x_{i}‐\bar{x}\right)}^{2}\sum_{i=1}^{n}{\left(b_{i}-\bar{b}\right)}^{2}}}$$ (3)

$$R_{\mathrm{POP}}=\frac{\sum_{i=1}^{n}\left(x_{i}‐\bar{x}\right)\left(c_{i}-\bar{c}\right)}{\sqrt{\sum_{i=1}^{n}{\left(x_{i}‐\bar{x}\right)}^{2}\sum_{i=1}^{n}{\left(c_{i}-\bar{c}\right)}^{2}}}$$ (4)

*R*_*DEM*_, *R*_*GDP*_, and *R*_*POP*_ respectively refer to the correlation coefficient between *x* (*x* denotes toponym density) and *a* (*a* denotes elevation), *b* (*b* denotes GDP), and *c* (*c* denotes population density). Furthermore, *x*_*i*_, a_i_, *b*_*i*_, and *c*_*i*_ respectively denote the toponym density, elevation, GDP, and population density of raster *i*.

#### Geo-Informatic Tupu

**Fig 3:**
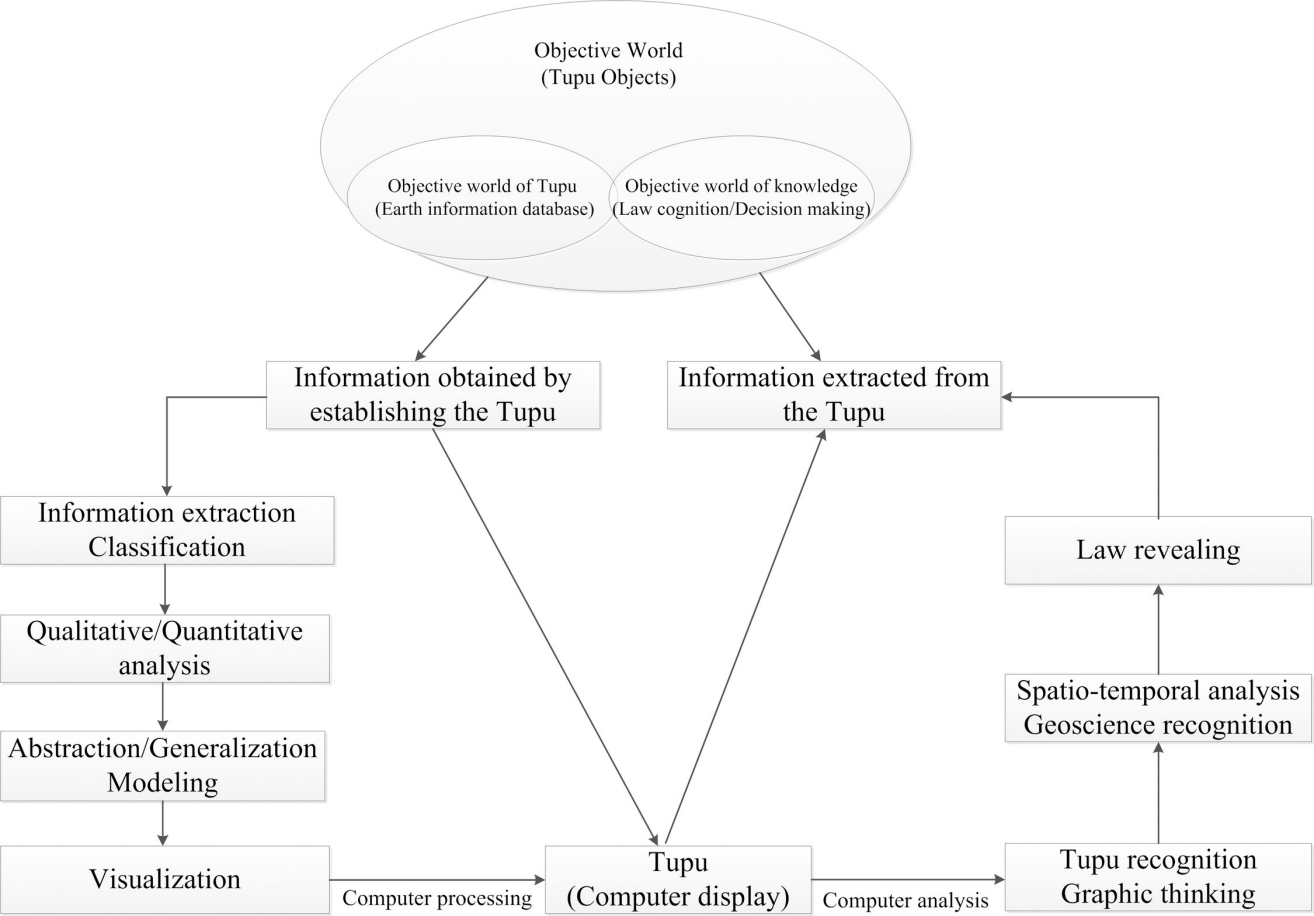
Generation and transmission mode of Geo-Informatic Tupu.

Geo-Informatic Tupu is a group of maps, curves, graphs, or tables arranged by certain indicators gradient rules or systematization laws, it utilizes the methods (such as GIS, RS, mathematical models, etc) to display spatial dynamic changes [42–47]. Although the theory and method of Geo-Informatic Tupu have been successfully applied to many branches of geoscience, it has not been applied to the study of spatio-temporal evolution of administrative toponyms or administrative divisions. In this paper, we used the generated maps and adopt the event-based state amendments model to establish the spatio-temporal database of administrative toponyms, then selected various indicators (such as toponym density, toponym naming time or life cycle, naming type, etc) to derive a series of spatio-temporal composite Geo-Informatic Tupu according to time sequence. Fig 3 shows the generation and transmission mode of Geo-Informatic Tupu [48]. In this paper, we adopted the spatio-temporal data model on account of shapefile and base state with amendments model [49] to establish the spatio-temporal database, the spatio-temporal evolution Tupu of county-level administrative toponyms densities in the eastern plains of China since the Sui was generated by using the KDE method, and extracted manually the naming time, naming basis and naming type information of county-level administrative toponyms since the Sui by relying on the expert knowledge from the *Dictionary of Chinese Historical Toponyms* [30], *The New Century Chinese City View* [31], *The Changes of Administrative Divisions in Chinese History* [37], *Encyclopedic Dictionary of Ancient and Modern Chinese Geographical Names* [38] and *China's Toponyms Anecdotes Dictionary* [39]. In view of the advantage of Geo-Informatic Tupu in analyzing and expressing the spatio-temporal changes, it is suitable to the study of spatio-temporal evolution characteristics of administrative toponyms in this paper.

## Results

### Analysis of the spatio-temporal evolutionary characteristics of the county-level administrative toponyms cultural landscape

#### Spatio-temporal evolutionary features of the county-level administrative toponyms cultural landscape

The statistical years are in keeping with *The Historical Atlas of China* [33] from Sui to Qing dynasties, used for the ROC is 1925 AD, and 2010 for the PRC.

**Fig 4:**
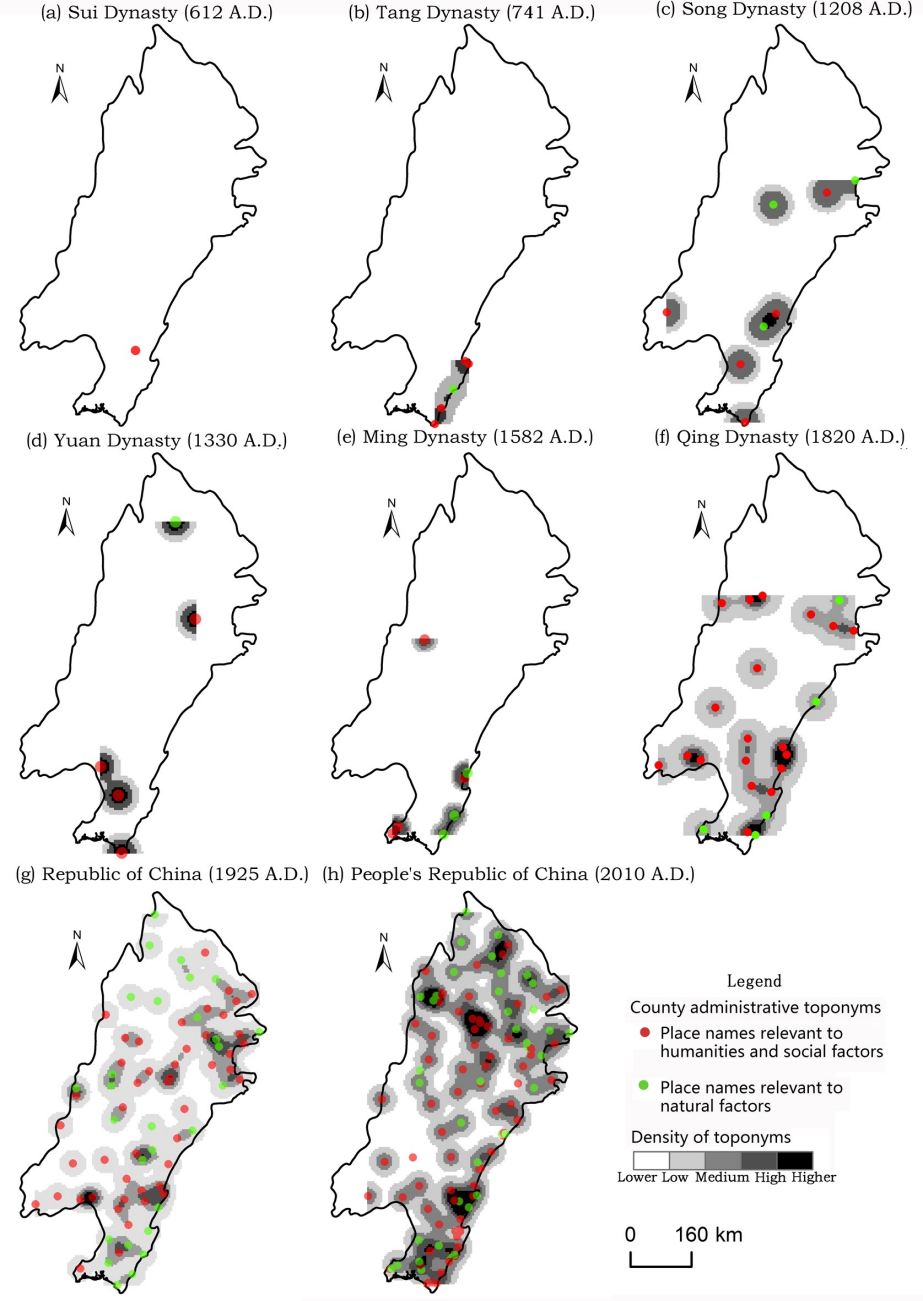
Spatio-temporal evolution of county-level administrative toponyms in the Northeast China Plain since the Sui dynasty.

As shown in Fig 4, the number of toponyms in the Northeast China Plain increased rapidly from the Qing dynasty onward, because of its role as the dynasty’s birthplace. Furthermore, the core of toponym densities gradually spread from south to north, finally causing the accumulation of areas at the intersections of the Lower Liao, Songhua, and Nen Rivers. Different types of county-level administrative toponyms are discussed in the next section.

**Fig 5:**
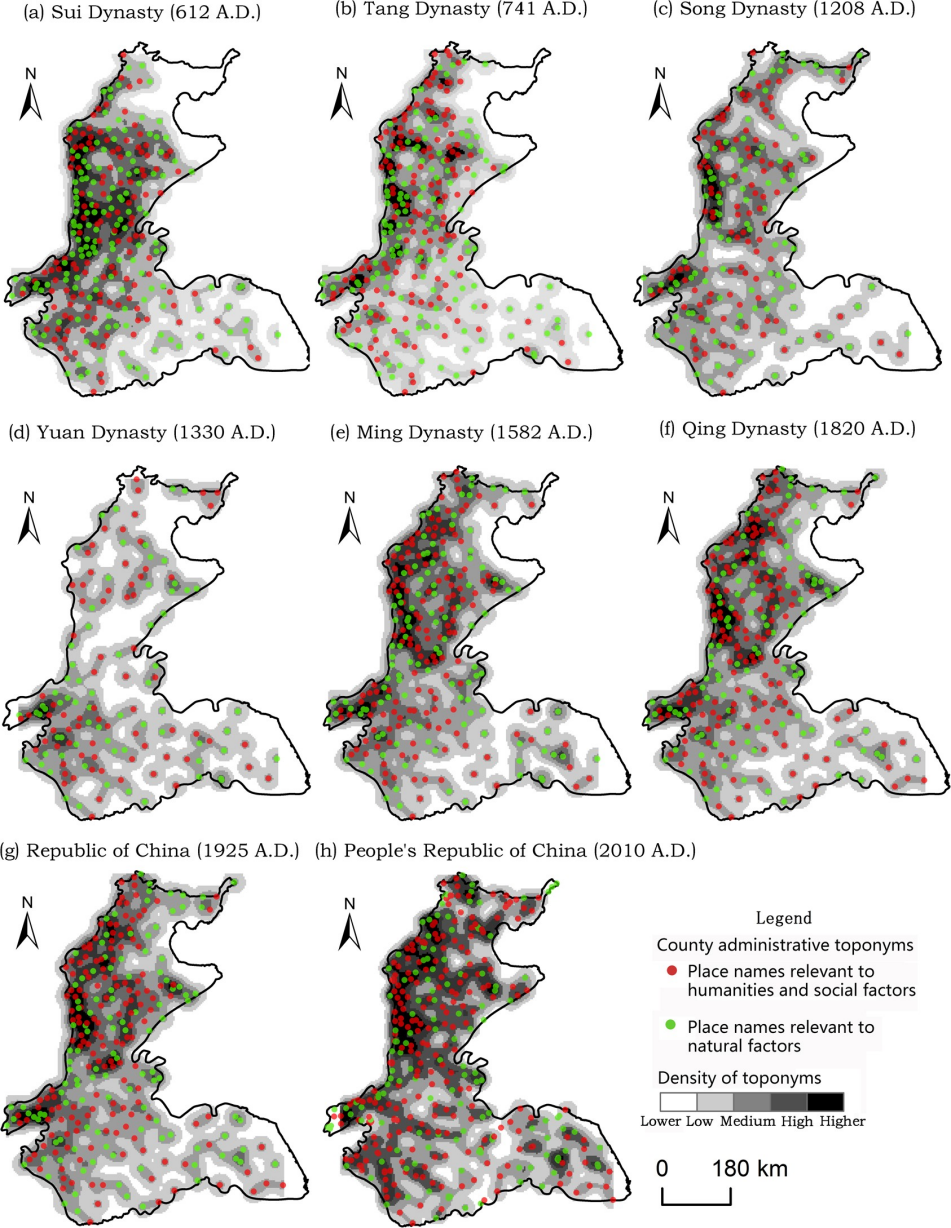
Spatio-temporal evolution of county-level administrative toponyms in the North China Plain since the Sui dynasty.

Fig 5 shows that the northwestern part of the North China Plain (i.e., the regions between the northern part of the Yellow River and eastern part of Taihang Mountain) always had a relatively high toponym density, except during the Yuan dynasty. Because ancient China moved its economic center from north to south during the Song dynasty, thereafter, especially during the Yuan, the density of toponyms gradually declined and the core area moved southward in the North China Plain. However, the Ming dynasty had a higher toponym density than the Yuan for complex reasons including the following. In terms of economic and demographic factors, the population and economic recovery increased during the Ming. Regarding political factors, the Ming dynasty’s Emperor Zhu Di moved the capital from Nanjing to Beijing, triggering the economic resurgence of the North China Plain. The density of toponyms did not change much after the Ming and Qing, because the Qing used the same administrative toponym system as the Ming.

**Fig 6:**
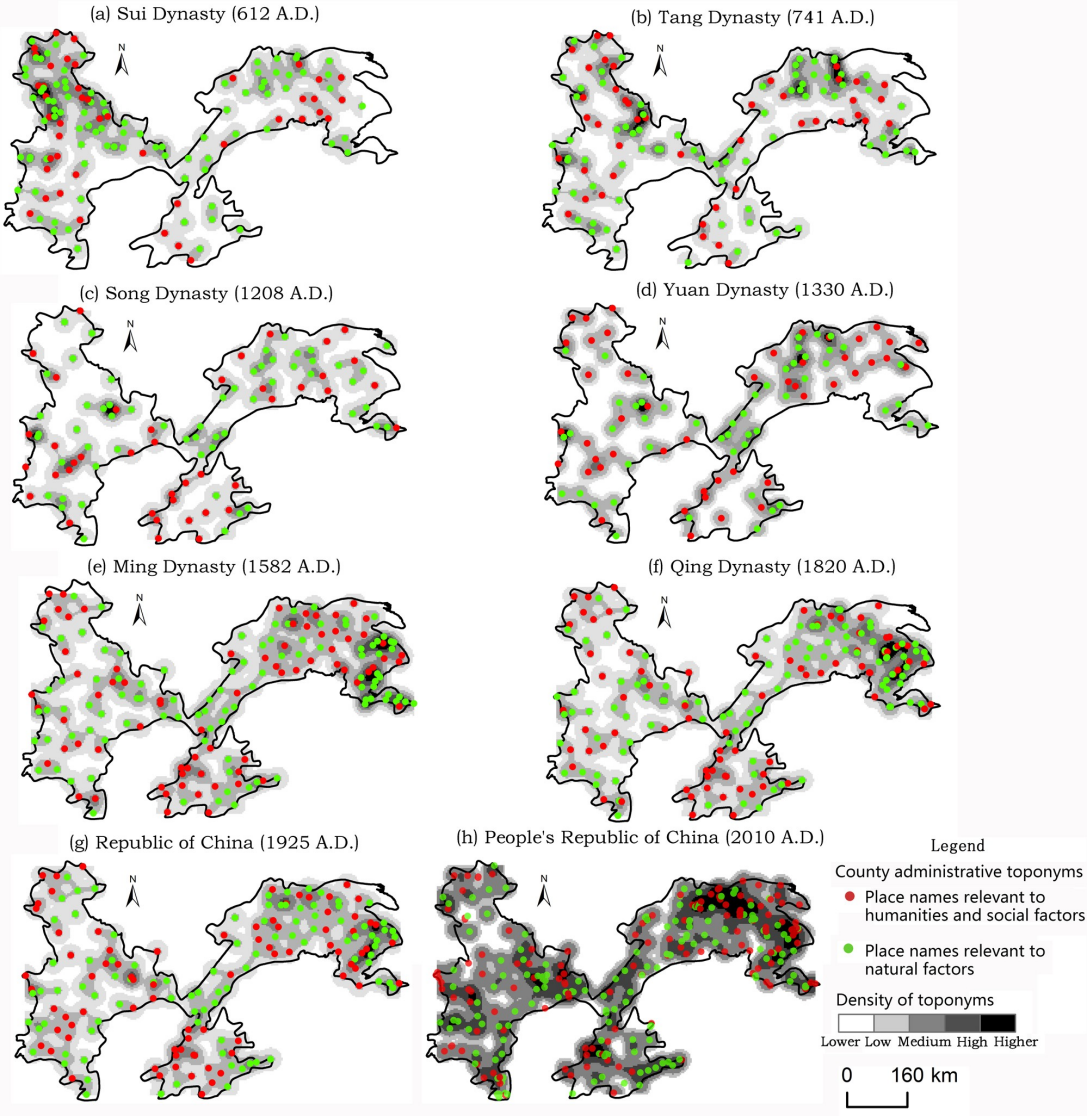
Spatio-temporal evolution of county-level administrative toponyms in the Yangtze Plain since the Sui dynasty.

The increasing prosperity and development of the maritime Silk Road during the Song and Yuan lead to rapid economic development in the southern part of China. In addition, Liujiagang Harbor, located in the Yangtze Plain, was the starting point of Zheng He’s voyage during the Ming. As shown in Fig 6, the center of county-level administrative toponyms began shifting eastward in the Yangtze Plain from the Song and Yuan. Today, urban agglomeration in the Yangtze River Delta, which is located in the Yangtze Plain, has lead it to become the most developed and urbanized region of China. Furthermore, the Yangtze River Delta has the densest concentration of county-level administrative toponyms in the Yangtze Plain.

As seen in Figs 4–6 and Table 1, the number of county-level administrative toponyms in the Northeast China Plain, North China Plain, and Yangtze Plain fluctuates based on the vicissitudes of dynasties. However, the trend in the number of toponyms is roughly increasing, mainly because new counties are being separated from old ones over time. Therefore, the PRC era has the most toponyms and the highest toponym density in the three plains and nationwide. Moreover, the number of toponyms is decreased in the Song and Yuan dynasties and the reasons are complex, may be relevant to the administrative division system and demographic/economic factors. For example, the Yuan dynasty implemented a multi-level compound administrative division system, and most of the prefecture-level administrative toponyms decreased to the county-level during the Ming and Qing dynasties [50].

**Table 1:** Statistical characteristics of county-level administrative toponyms.

Period	Number of toponyms				Average toponym density (per 10^4^ km^2^)			
	Northeast China Plain	North China Plain	Yangtze Plain	Whole nation	Northeast China Plain	North China Plain	Yangtze Plain	National average
Sui	1	279	132	1271	0.03	8.51	5.01	0.92
Tang	5	284	119	1575	0.16	8.67	4.51	1.13
Song	8	206	98	1129	0.26	6.29	3.72	0.81
Yuan	5	142	105	1123	0.16	4.33	3.98	0.80
Ming	7	267	178	1427	0.23	8.15	6.75	1.03
Qing	24	270	183	1549	0.79	8.24	6.94	1.11
ROC	76	280	195	2044	2.49	8.54	7.39	1.79
PRC	125	388	298	2856	4.10	11.84	11.30	2.98

Furthermore, since the Sui dynasty, the toponym densities in the three plains are almost all greater than the national average density in the corresponding period. In particular, the toponym densities in the North China Plain and Yangtze Plain are more than triple the national average during the corresponding periods since the Sui.

#### Naming time of county-level administrative toponyms

**Fig 7:**
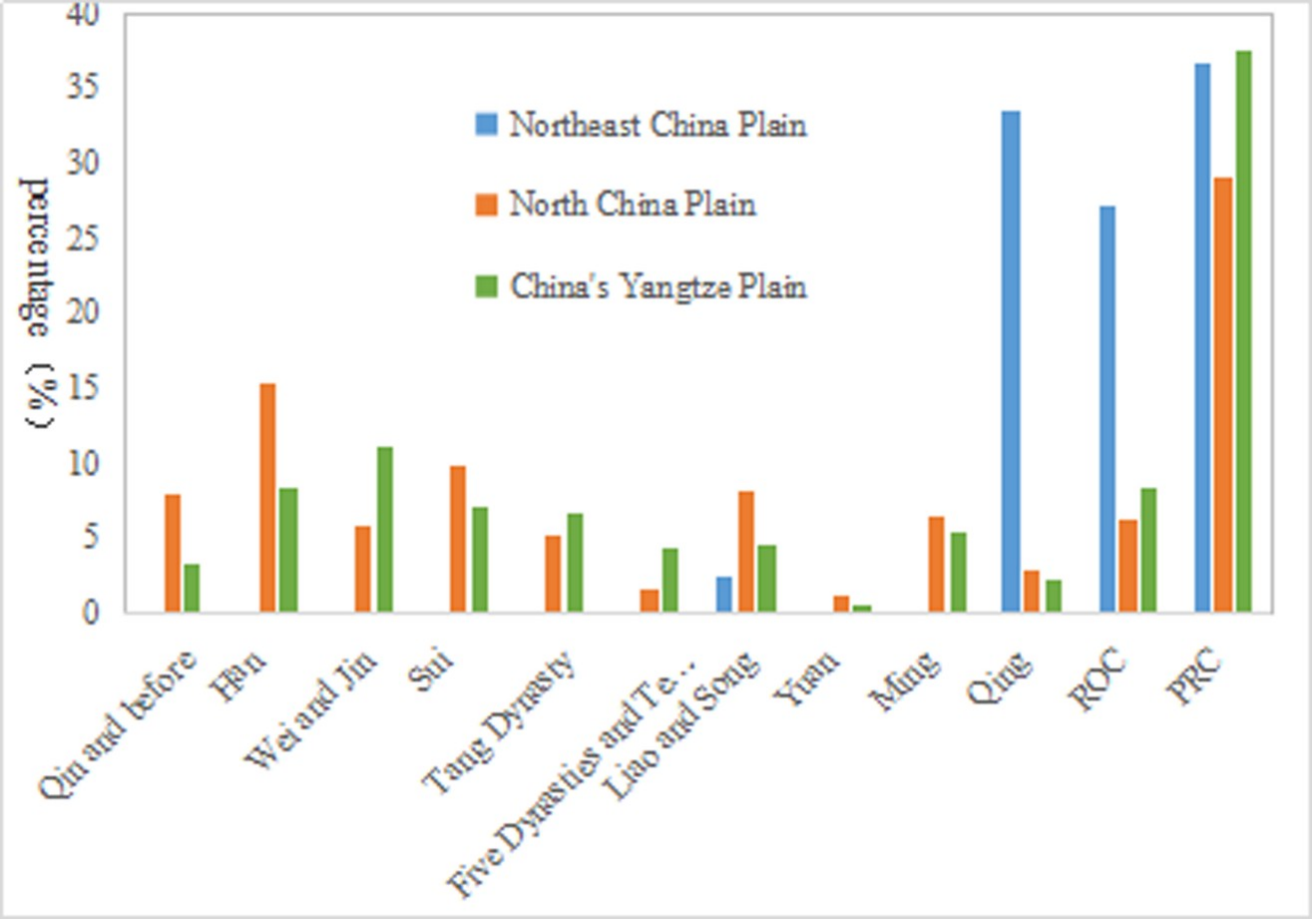
Distribution characteristics of naming time of county-level administrative toponyms in 2010.

Fig 7 indicates that in 2010, the naming time of county-level administrative toponyms in the Northeast China Plain were comparatively concentrated, but relatively scattered in the North China Plain and Yangtze Plain. However, naming during the PRC period has the highest proportion in all three plains. Specifically, over 97% of county-level administrative toponyms in the Northeast China Plain were named since the Qing (125 in total). The Qing is the second-most common, accounting for more than 34%. Furthermore, 46% of county-level administrative toponyms in the North China Plain have had their names for more than 1,000 years (388 in total), with most coming from the Han. In the Yangtze Plain, 41% of county-level administrative toponyms have had their names for more than 1,000 years (298 in total), with the Wei-Jin making up the second-largest proportion.

The evolution of county-level administrative toponyms can also indicate local landscape changes. For example, Hekou District in the North China Plain was named in 1984 based on its location at the estuary of the Yellow River. However, Hekou District is located on what was once a sea area. People originally settled there from the Qing onward. Qidong City in the Yangtze Plain was named in 1989, since it was a developing eastern coastal area. Qidong City was located by the estuary of the Yangtze River, which was also once a sea area. It initially emerged as a shoal made by the sands of the Yangtze River [39].

#### Naming basis of county-level administrative toponyms

After analyzing the naming basis of county-level administrative toponyms, we inferred the factors influencing their spatio-temporal evolution. Based on scholarly research achievements [3, 39, 51], we divided the 811 county-level administrative toponyms into 11 categories according to the characteristics of their proper names in China’s plains areas (Table 2). For example, the naming type of Dangshan County is relevant to mountains, Lishui District relevant to hydrological features, Yandu District relevant to products, Wanli District relevant to terrain, Ningxiang County relevant to blessings, Dantu District relevant to historical figures and events, etc. Moreover, the naming types relevant to mountains, hydrological features, products, and terrain belong to natural factors, while relevant to orientation, blessings, ancient relics, historical figures and events, surnames, taboos, and other forms belong to human/social factors.

**Table 2:** Distribution characteristics of naming types of county-level administrative toponyms in 2010.

Naming type	Northeast China Plain		North China Plain		Yangtze Plain	
	Count	(%)	Count	(%)	Count	(%)
Relevant to mountains	9	7.2	24	6.2	39	13.1
Relevant to hydrological features	30	24.0	94	24.2	102	34.2
Relevant to products	6	4.8	10	2.6	8	2.7
Relevant to terrain	4	3.2	15	3.9	6	2.0
Relevant to orientation	13	10.4	37	9.5	18	6.0
Relevant to blessings	19	15.2	70	18.0	38	12.8
Relevant to ancient relics	22	17.6	53	13.7	44	14.8
Relevant to historical figures and events	7	5.6	53	13.7	26	8.7
Relevant to surnames	3	2.4	8	2.1	5	1.7
Relevant to taboos	0	0	6	1.6	5	1.7
Other forms	12	9.6	18	4.6	7	2.3
Total	125	100	388	100	298	100

Table 2 clarifies that in 2010, county-level administrative toponyms relevant to hydrological features constituted the largest proportion in the Northeast China Plain, North China Plain, and Yangtze Plain. This demonstrates that China’s plains areas were relatively abundant in water resources during historical periods; for example, the Liao, Yellow, Huai, and Yangtze Rivers all flow across these three plains. Historically, people tended to settle nearby a water source. Next, county-level administrative toponyms related to ancient relics constitute a relatively large proportion, indicating that the plains areas have a long history and abundant cultural deposits. Then, county-level administrative toponyms relevant to blessings account for the second-largest percentage in the North China Plain. This could be attributed to the frequent hazards caused by the Yellow River’s shifting course, floods, and overflows, which made people seek peaceful and prosperous lives using toponyms containing good wishes. In addition, based on the existence of ethnic minority regimes during historical periods, more toponyms in the Northeast China Plain are derived from the Mongolian or Manchu languages than in the other two plains.

Furthermore, the number of county-level administrative toponyms relevant to mountains and hydrological features in the Northeast China Plain, North China Plain, and Yangtze Plain in 2010 accounted for more than 30% of all toponyms, with the largest proportion in the Yangtze Plain and lowest in the North China Plain. This demonstrates that natural landscapes with mountains and rivers greatly influence the naming of administrative units in China’s plains areas, as in the rest of China. Besides, we used Python to write the statistical word frequency code of administrative toponyms since the Sui, and ran the results with the help of Python 2.7. The results show that the words of “Yang” (the south of mountain or north of river), “Shan” (mountain), and “Jiang” (river) always had high frequencies. Especially, “Shan” had the highest frequency in 2010. According to Professor Shi Nianhai, the naming of administrative units focused on geographic factors, and toponyms named after natural landscapes such as mountains and rivers were universal and stable [51]. Therefore, to further analyze the correlations between naming rules and natural factors, we sorted the statistics of county-level administrative toponyms relevant to natural factors since the Sui (Table 3).

**Table 3:** Statistical characteristics of county-level administrative toponyms relevant to natural factors since the Sui dynasty.

Period	Northeast China Plain		North China Plain		Yangtze Plain		Total	
	Count	(%)	Count	(%)	Count	(%)	Count	(%)
Sui	0	0	151	54.1	89	67.4	240	58.3
Tang	1	20.0	140	49.3	75	63.0	216	52.9
Song	3	37.5	98	47.6	57	58.2	158	50.6
Yuan	1	20.0	65	45.8	59	56.2	125	49.6
Ming	3	42.9	116	43.4	100	56.2	219	48.5
Qing	5	20.8	111	41.1	98	53.6	214	44.9
ROC	28	36.8	109	38.9	103	52.8	240	43.6
PRC	43	34.4	141	36.3	153	51.3	337	41.6

As shown in Table 3, the percentages of county-level administrative toponyms in the North China Plain and Yangtze Plain relevant to natural factors has been declining since the Sui, as have those for the total of three plains in spite of always staying above 40%. Conversely, the percentage of toponyms concerned with human/social factors has increased. This is also evident in Figs 4–6. Moreover, 58.4% of the total toponyms in the three plains were relevant to human/social factors in 2010. However, the Northeast China Plain does not demonstrate the same rule, because of the small number of samples and influence of ethnic minority languages.

Therefore, we contend that natural factors (such as mountains and hydrological features) always played a vital role in the naming of county-level administrative toponyms since the Sui, yet human/social factors will play a more critical role than natural factors in the evolution of the naming basis of the county-level administrative toponyms cultural landscape in China’s eastern plains areas.

### Factors influencing the spatio-temporal evolution of the county-level administrative toponyms cultural landscape

#### Factors influencing spatial differentiation

**Fig 8:**
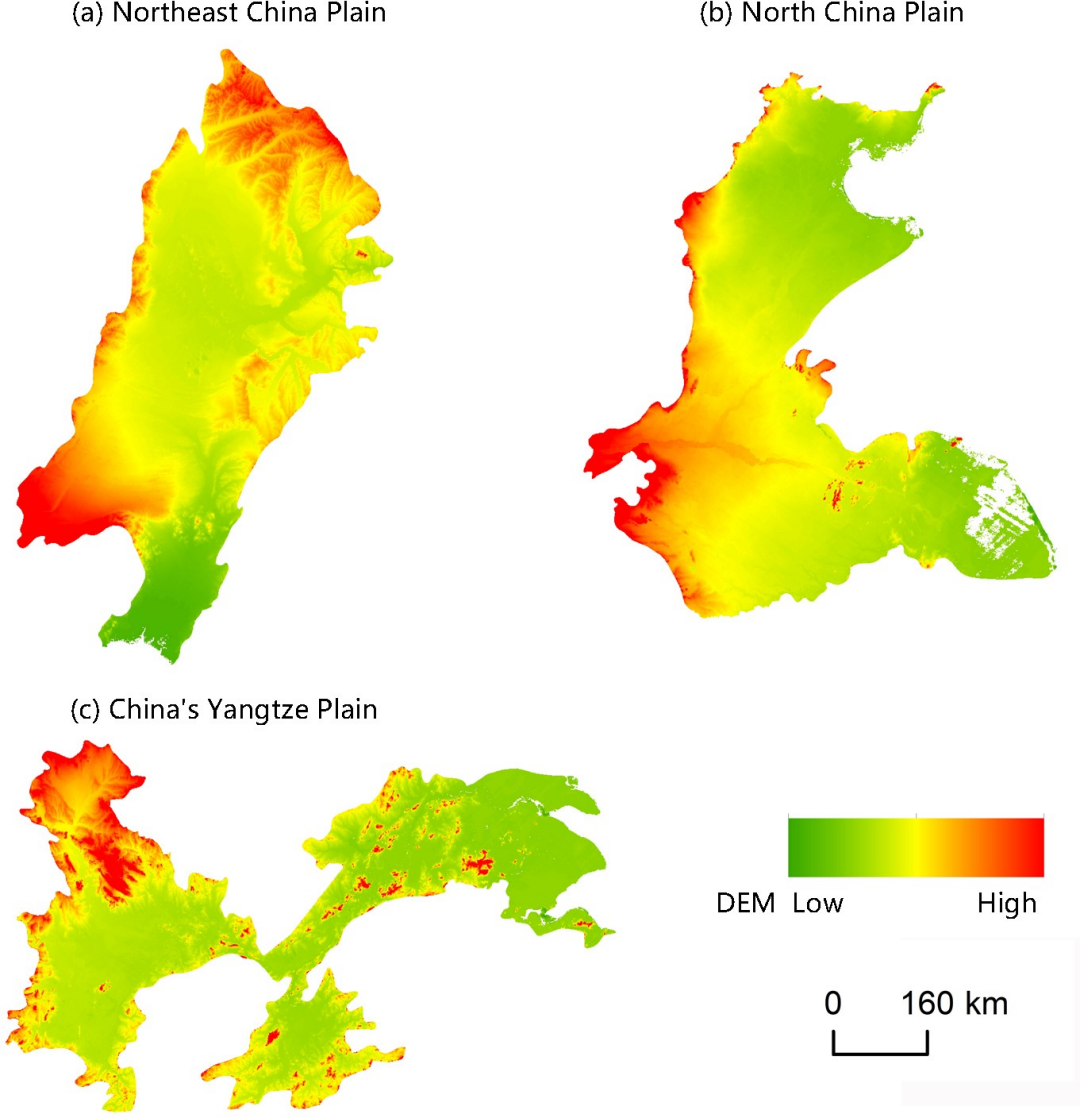
DEM data of the case study areas. DEM data at a resolution of 30 m were provided by the RESDC.

**Fig 9:**
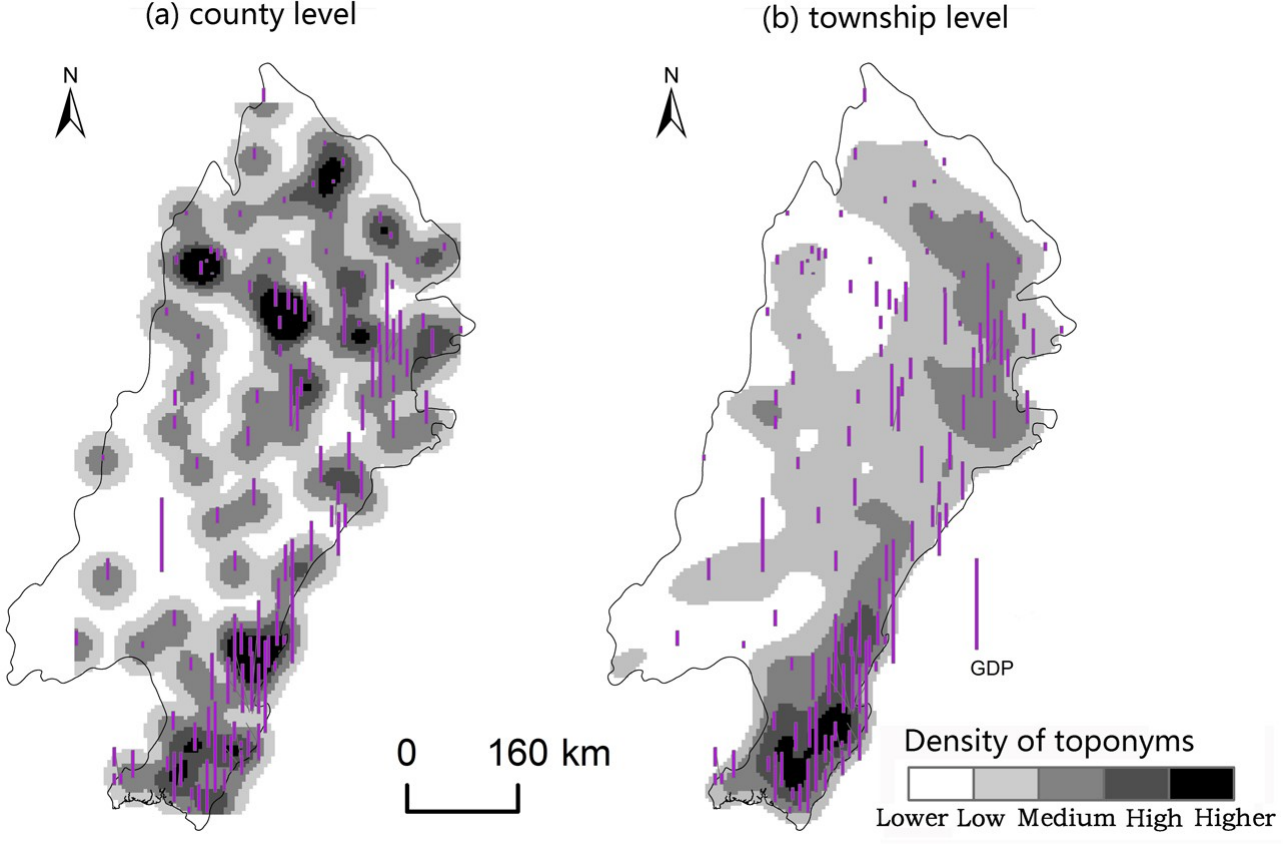
Spatial distribution of toponym density and county GDP of the Northeast China Plain. The statistical data on China’s GDP in 2010 (Figs 8, 10, 12) were obtained from the RESDC.

**Fig 10:**
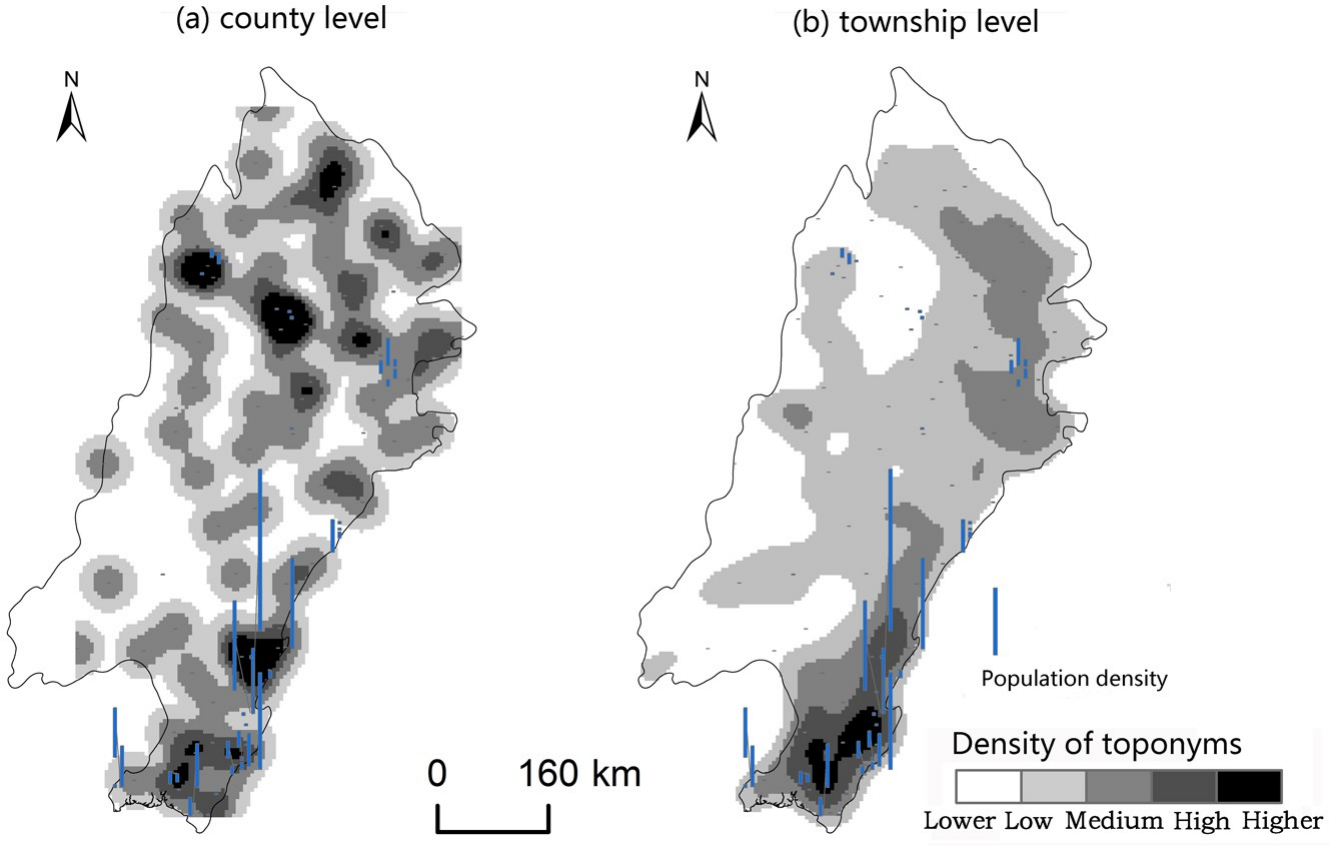
Spatial distribution of toponym density and county population density in the Northeast China Plain. Population data at the county level (Figs 9, 11, 13) were obtained from the 6^th^ Census of China (2010).

**Fig 11:**
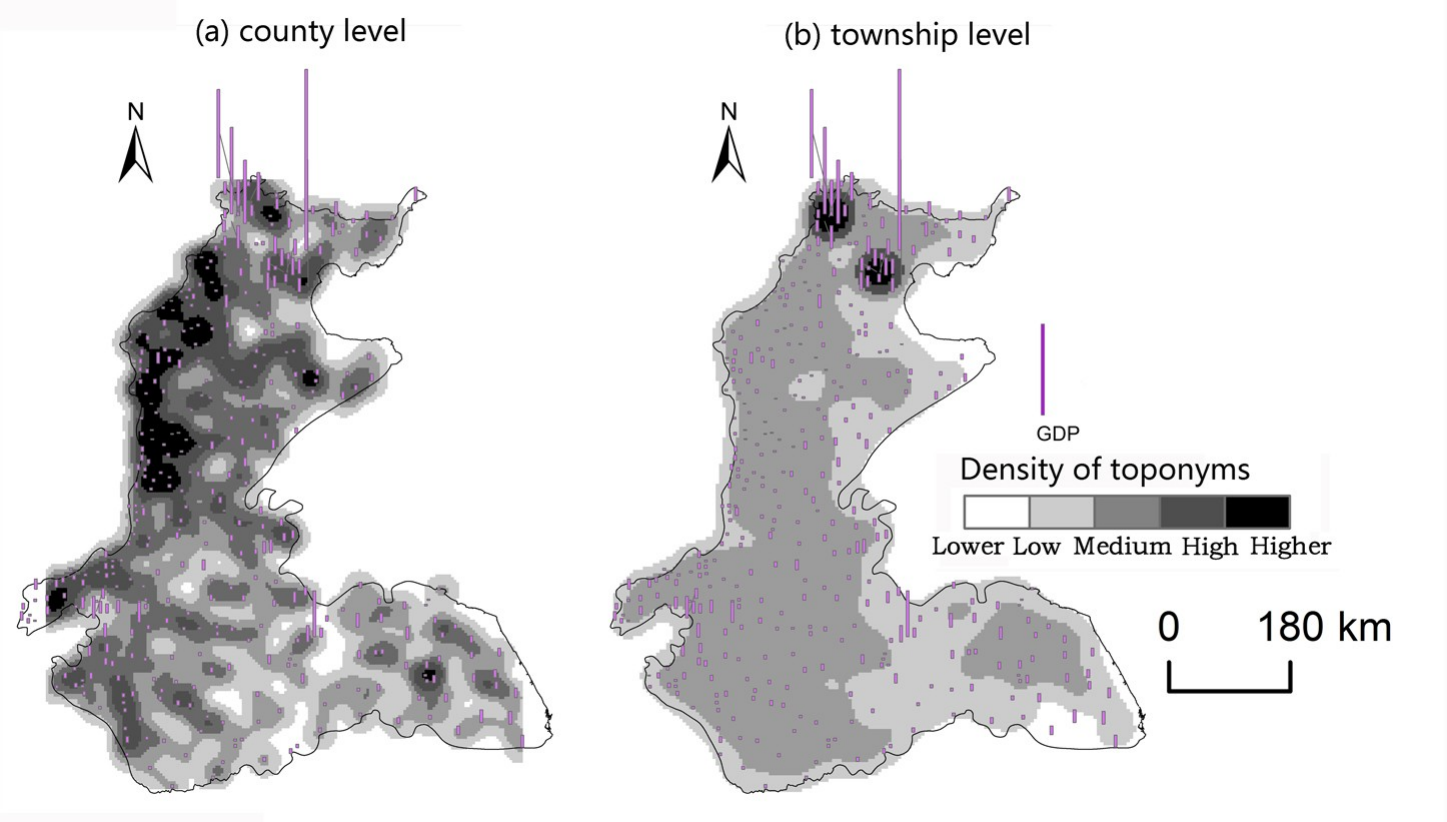
Spatial distribution of toponym density and county GDP of the North China Plain.

**Fig 12:**
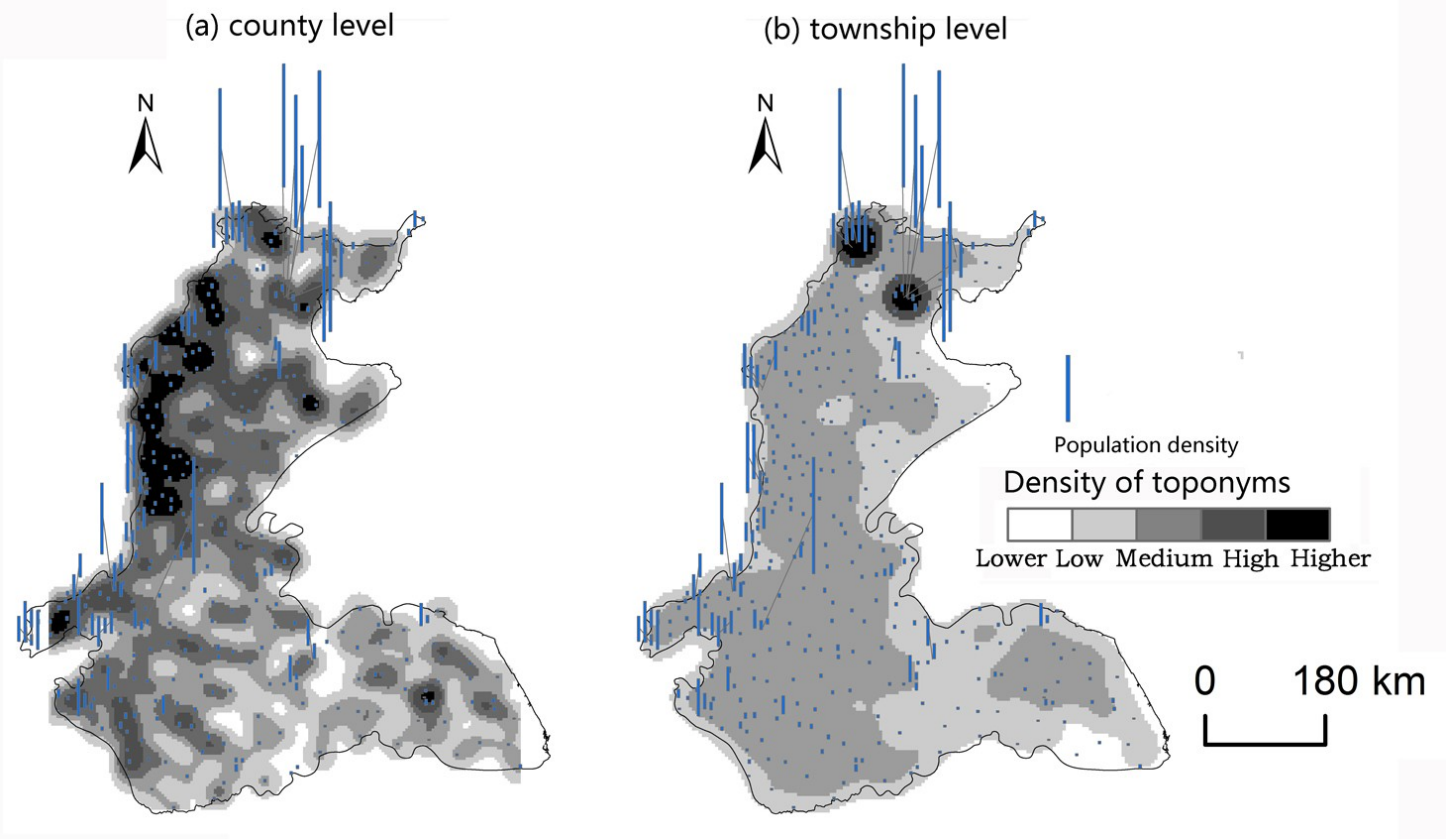
Spatial distribution of toponym density and county population density in the North China Plain.

**Fig 13:**
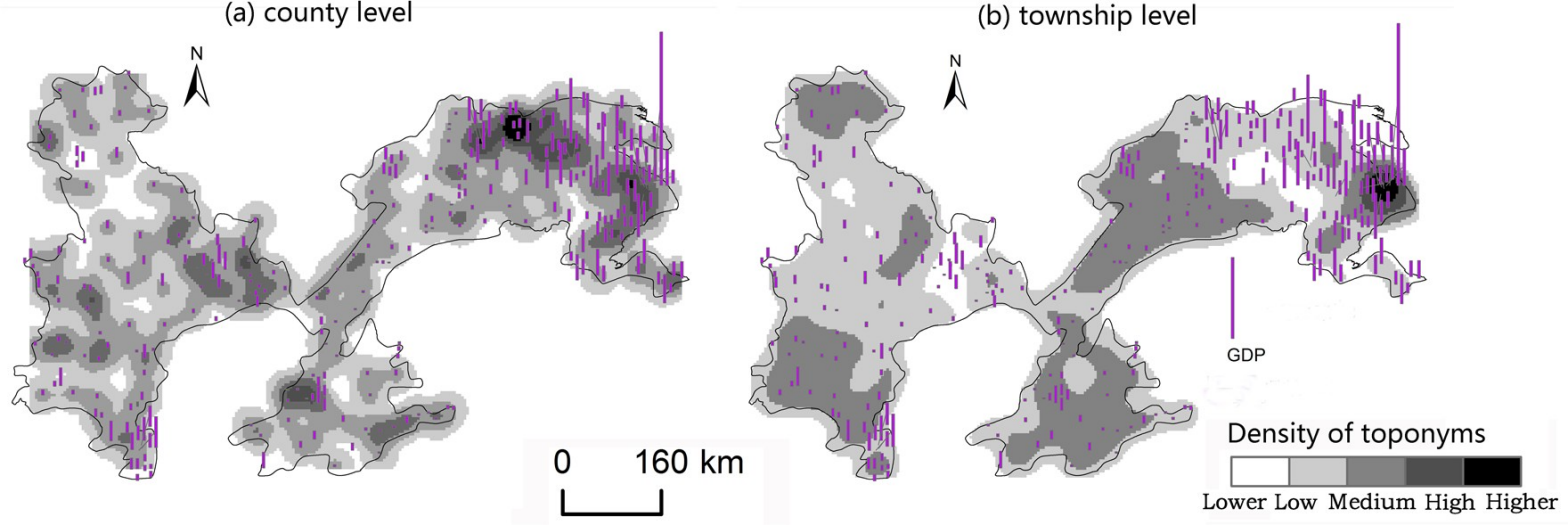
Spatial distribution of toponym density and county GDP of the Yangtze Plain.

**Fig 14:**
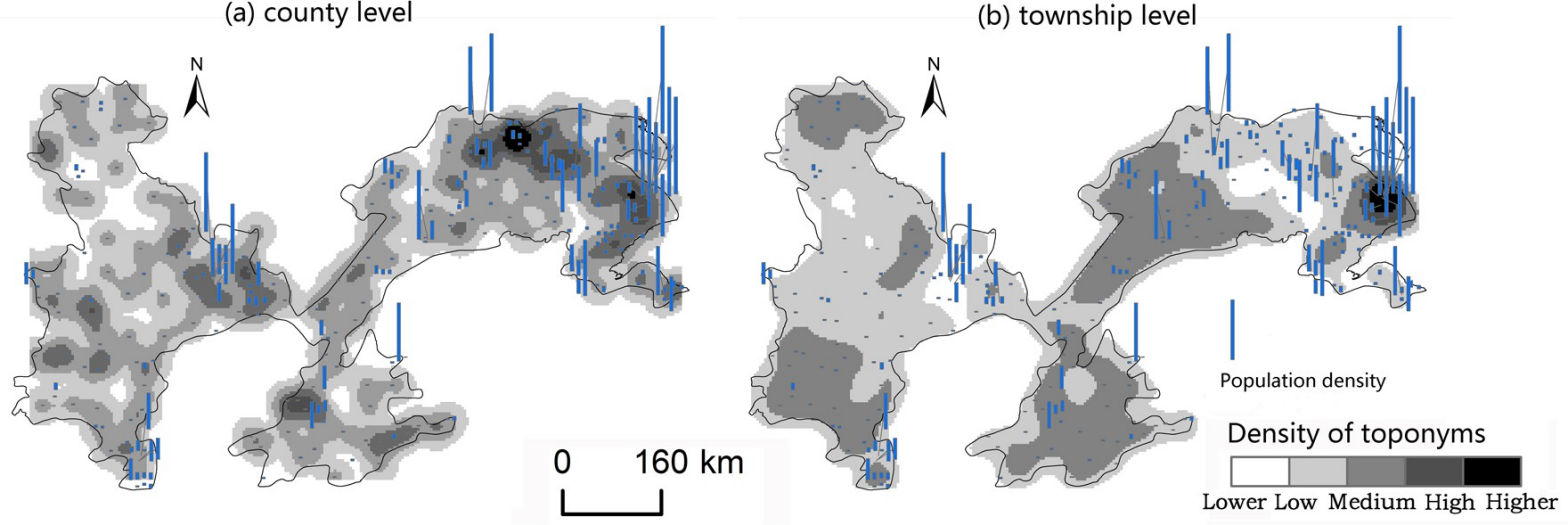
Spatial distribution of toponym density and county population density in the Yangtze Plain.

Changes caused by the allocation and distribution of political districts are dynamic indicators of economic prosperity and population changes [52]. Generally, a more developed district has a larger number of administrative toponyms, and vice versa. To explore the influencing factors of the spatial differentiation of county-level administrative toponyms in the plains areas of China, we analyzed the coupling relationships between toponyms and natural, economic, and demographic factors. First, we demonstrated the spatial distribution of these elements by using data on toponym density, DEM, county GDP, and county population density in 2010, as shown in Figs 8–14. Subsequently, we also conducted a quantitative analysis of the spatial correlations between toponyms and natural, economic, and demographic factors at the grid level using grid data on China’s GDP and population in 2010. The correlation coefficients listed in Table 4 were calculated using formulae (2)–(4).

**Table 4:** Correlation coefficients between county-level administrative toponym density and DEM, GDP, and population density.

Plain	R_DEM_	R_GDP_	R_POP_
Northeast China Plain	-0.73	0.90*	0.92*
North China Plain	-0.66	0.78*	0.81*
Yangtze Plain	-0.60	0.88**	0.90**

* indicated the value was significant at the 0.05 level (2-tailed).

** indicated the value was significant at the 0.01 level (2-tailed). The grid data on China’s population and GDP in 2010 (at a 1-km gridded resolution) were provided by the RESDC.

Figs 8–14 show the spatial distributions of toponym density and DEM, county GDP, and county population density in the Northeast China Plain, North China Plain, and Yangtze Plain. However, these figures can only demonstrate spatial correlations between toponyms and natural, economic, and demographic factors in a simple and intuitive way. Consequently, we calculated the correlation coefficients between the density of county-level administrative toponyms and DEM, GDP, and population density at the grid level using formulae (2)–(4), as shown in Table 4.

The absolute values of the Pearson correlation coefficients shown in Table 4 are all larger than 0.50, indicating that county-level administrative toponym density is correlated with DEM, GDP, and population density. Moreover, the correlation coefficients between toponym density and DEM were negative, meaning that regions in the plains areas at a higher elevation had fewer administrative toponyms. Conversely, the correlation coefficients between toponym density and GDP and population density were positive; thus, regions with higher GDPs or population densities had more toponyms. In addition, the results showed that the correlation degree between toponym density and population density was the highest in these three plains, and that between toponym density and DEM the lowest. This suggests that population has a greater effect on the distribution of toponyms than altitude. Furthermore, Table 4 shows that the correlation degree between toponym density and DEM in the Northeast China Plain was the highest, and lowest for the Yangtze Plain. The correlation degree between toponym density and GDP and population density in the Northeast China Plain was the highest, and lowest in the North China Plain.

The correlation coefficients in Table 4 were calculated by using the grid data of county-level administrative toponym density, DEM, GDP, and population density, then Table 5 demonstrates that we used the statistical data of GDP, and population density to evaluate the applicability of these correlations. Considering the data characteristics, the county-level administrative toponym density was the ratio between the number of county-level administrative toponyms and the area of the county-level administrative district, the DEM data was the mean value of grid data in the county-level district. Because the boundaries of the three plains were across some county-level districts, the data of GDP, and population density in these county-level districts could not be simply calculated by the area ratios. Based on the research achievement from some scholars [53], we calculated the GDP, and population density in these county-level districts by using the the spatial distribution rules of the relief degree of land surface at county level and its correlation between GDP and population density. In addition, we also calculated the correlation coefficients between township-level toponym density and DEM, GDP, and population density to verify the applicability of county-level correlations by downscaling analysis (Table 5), and also got similar results: the correlation degree between toponym density and population density was the highest in these three plains, and that between toponym density and DEM the lowest, the correlation degree between toponym density and DEM in the Northeast China Plain was the highest, and lowest for the Yangtze Plain.

**Table 5:** Correlation coefficients between administrative toponym density and DEM, GDP, and population density.

Plain	County-level administrative toponym density			Township-level administrative toponym density		
	R_DEM_	R_GDP_	R_POP_	R_DEM_	R_GDP_	R_POP_
Northeast China Plain	-0.68	0.75*	0.89**	-0.78	0.80*	0.85*
North China Plain	-0.62	0.81*	0.83*	-0.59	0.89*	0.91*
Yangtze Plain	-0.56	0.76**	0.85**	-0.54	0.93**	0.95**

* indicated the value was significant at the 0.05 level (2-tailed).

** indicated the value was significant at the 0.01 level (2-tailed). The GDP data in 2010 at the county level were from the RESDC and population data at the county level from the 6^th^ Census of China (2010).

In conclusion, the spatial distribution maps (see Figs 8–14) and correlation coefficients (Tables 4 and 5) illustrate the factors influencing county-level administrative toponym spatial differentiation. The results intuitively showed that DEM, GDP, and population density were key elements in the spatial differentiation of toponyms cultural landscape in China’s plains areas, although population density played a greater role in these changes than natural factors.

#### Factors influencing temporal evolution

Considering the availability of historical data, we only analyzed the coupling relationship between the number of county-level administrative toponyms and historical temperatures, and between the number of county-level administrative toponyms and historic population levels. For the temperature data, we referred to Zhu’s temperature change curve for 5,000 years in China [54].

**Fig 15:**
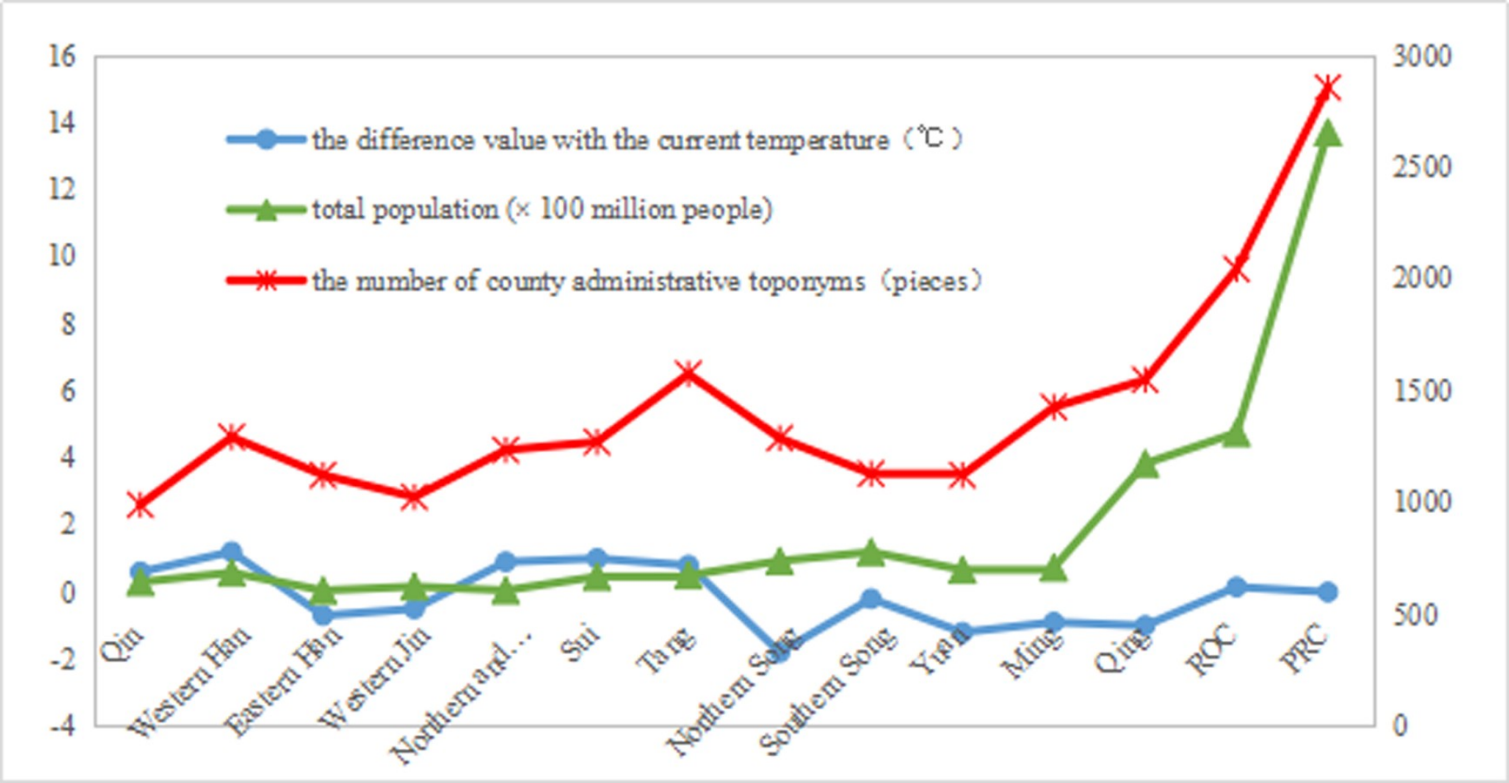
Trend chart of toponym, population, and temperature changes. The temperature values in Fig 15 are the change values with the current temperature. For historical population data, we referred to the statistical data from the National Earth System Science Data Sharing Infrastructure, National Science & Technology Infrastructure of China, and made some adjustments.

Fig 15 shows that the change in the number of toponyms is consistent with temperature changes from the Qin to the ROC and changes in the population size since the Ming. Furthermore, toponym density demonstrates a relative growth since the Ming (Table 1). We contend that temperature changes were important in historic toponym changes, and population changes have influenced toponym changes over the last 400 years in China.

The factors influencing the temporal evolution of county-level administrative toponyms are complex and sometimes have a delayed effect. For example, although county-level administrative districts are the most stable and basic administrative regions in China’s history, the ranks of county-level administrative toponyms range from the second to the fifth. Political factors mainly affect the evolution of county-level administrative toponyms ranks. Specifically, county-level administrative toponyms are often in different ranks in the administrative toponym systems of different dynasties. For example, one might belong to the second level in the Qin and Western Han dynasties; third level in the Eastern Han and Southern and Northern dynasties; third, fourth, or fifth levels in the Yuan; third or fourth levels in the Ming; and so on. Some scholars [55, 56] consider the fall of a dynasty a complicated event, and while temperature and changes in dynasties may be correlated, simple correlation statistics may not be appropriate for complex paleoclimates and histories. Thus, we conducted a preliminary study of the factors influencing the spatio-temporal evolution of the county-level administrative toponyms cultural landscape based on the availability of historical data.

## Conclusions

This paper intuitively demonstrated the spatial-temporal characteristics and causes of changes in the county-level administrative toponyms cultural landscape in China’s eastern plains areas through the application of GIS spatial analysis, Geo-Informatic Tupu, KDE method, and correlation coefficients. The conclusions are summarized as follows.

1. In China’s eastern plains areas, the number of administrative toponyms has roughly increased over time since the Sui, the number of toponyms is decreased in Song and Yuan dynasties.
2. The results strongly indicate that high population density and exploitation intensity can trigger spatial and temporal changes to administrative toponyms. This can also be applied in interpreting the moving of China’s political center, economic barycenter, and population gravity center in historical periods.
3. In 2010, the naming time distribution of county-level administrative toponyms in the Northeast China Plain was comparatively concentrated, but relatively scattered in the North China Plain and Yangtze Plain. However, the highest proportion of naming time in all three plains was during the PRC period.
4. County-level administrative toponyms related to mountains and hydrological features accounted for more than 30% of all toponyms in 2010. However, the percentage of total county-level administrative toponyms in the three plains related to natural factors has been declining since the Sui. Conversely, the percentage of toponyms concerned with human/social factors is increasing. Therefore, we contend that natural factors (such as mountains and hydrological features) always played a vital role in the naming of county-level administrative toponyms since the Sui, yet human/social factors will play a more critical role than natural factors in the evolution of the naming basis of the county-level administrative toponyms cultural landscape in China’s eastern plains areas.
5. To explore the factors influencing the spatial differentiation of county-level administrative toponyms cultural landscape, we further analyzed the correlations between it and DEM, GDP, and population density. We negatively correlated county-level toponym density and DEM in all three plains, and positively correlated toponym density with GDP and population density. Furthermore, the correlation degree between toponym density and population density was the highest, and that between toponym density and DEM the lowest. We preliminarily explored the factors influencing the temporal evolution of the county-level administrative toponyms cultural landscape by analyzing the change trends of toponyms, populations, and temperatures since the Qin. We confirmed that the change trend regarding toponym numbers was consistent with temperature changes from the Qin to the ROC and with population size changes since the Ming. Furthermore, toponym density demonstrated a relative growth since the Ming.

The approach used in this paper had limitations regarding the data and methodology. For example, our research on the spatio-temporal distribution characteristics of toponyms in historical periods was confined to the county level, and smaller dimensions such as the township and village levels from the Sui to the ROC were not included in the study. Furthermore, we only analyzed the spatio-temporal features of administrative toponym changes and influencing factors in the plains areas. Thus, more researches are required on the evolutionary characteristics of toponyms in different geomorphic settings such as mountains, hills, plateaus, and basin regions, and other factors which could affect the spatio-temporal features of administrative toponyms.

Nevertheless, this study breaks through the restriction of administrative regions to reveal the rules and causes of the spatio-temporal evolutionary characteristics of toponyms by using China’s eastern plains areas as a study object, because it features the most intimate correlation between humans and the environment. Importantly, through a quantitative analysis and visualization of the correlations between toponyms and natural, economic, and demographic factors through Geo-Informatic Tupu, this study provides a new perspective for the future study of toponyms.

## Supporting information

S1 Fig Distribution map of county-level administrative toponyms with the time of being named from 1,000 years ago. (TIF)

S2 Fig Map of the case study areas. (TIF)

S3 Fig Generation and transmission mode of Geo-Informatic Tupu. (TIF)

S4 Fig Spatio-temporal evolution of county-level administrative toponyms in the Northeast China Plain since the Sui dynasty. (TIF)

S5 Fig Spatio-temporal evolution of county-level administrative toponyms in the North China Plain since the Sui dynasty. (TIF)

S6 Fig Spatio-temporal evolution of county-level administrative toponyms in the Yangtze Plain since the Sui dynasty. (TIF)

S7 Fig Distribution characteristics of naming time of county-level administrative toponyms in 2010. (TIF)

S8 Fig DEM data of the case study areas. (TIF)

S9 Fig Spatial distribution of toponym density and county GDP of the Northeast China Plain. (TIF)

S10 Fig Spatial distribution of toponym density and county population density in the Northeast China Plain. (TIF)

S11 Fig Spatial distribution of toponym density and county GDP of the North China Plain. (TIF)

S12 Fig Spatial distribution of toponym density and county population density in the North China Plain. (TIF)

S13 Fig Spatial distribution of toponym density and county GDP of the Yangtze Plain. (TIF)

S14 Fig Spatial distribution of toponym density and county population density in the Yangtze Plain. (TIF)

S15 Fig Trend chart of toponym, population, and temperature changes. (TIF)

S1 Table Statistical characteristics of county-level administrative toponyms. (PDF)

S2 Table Distribution characteristics of naming types of county-level administrative toponyms in 2010. (PDF)

S3 Table Statistical characteristics of county-level administrative toponyms relevant to natural factors since the Sui dynasty. (PDF)

S4 Table Correlation coefficients between county-level administrative toponym density and DEM, GDP, and population density. (PDF)

S5 Table Correlation coefficients between administrative toponym density and DEM, GDP, and population density. (PDF)

S1 File Chinese population census data of 2010. (RAR)

S2 File Grid data of China's population and GDP in 2010. (RAR)
