# Supplementary figures and images for: Spatial-temporal characteristics and causes of changes to the county-level administrative toponyms cultural landscape in the eastern plains of China
Source: PLoS One. 2019 May 28;14(5):e0217381. doi: 10.1371/journal.pone.0217381 (PMC6538164; doi:10.1371/journal.pone.0217381)

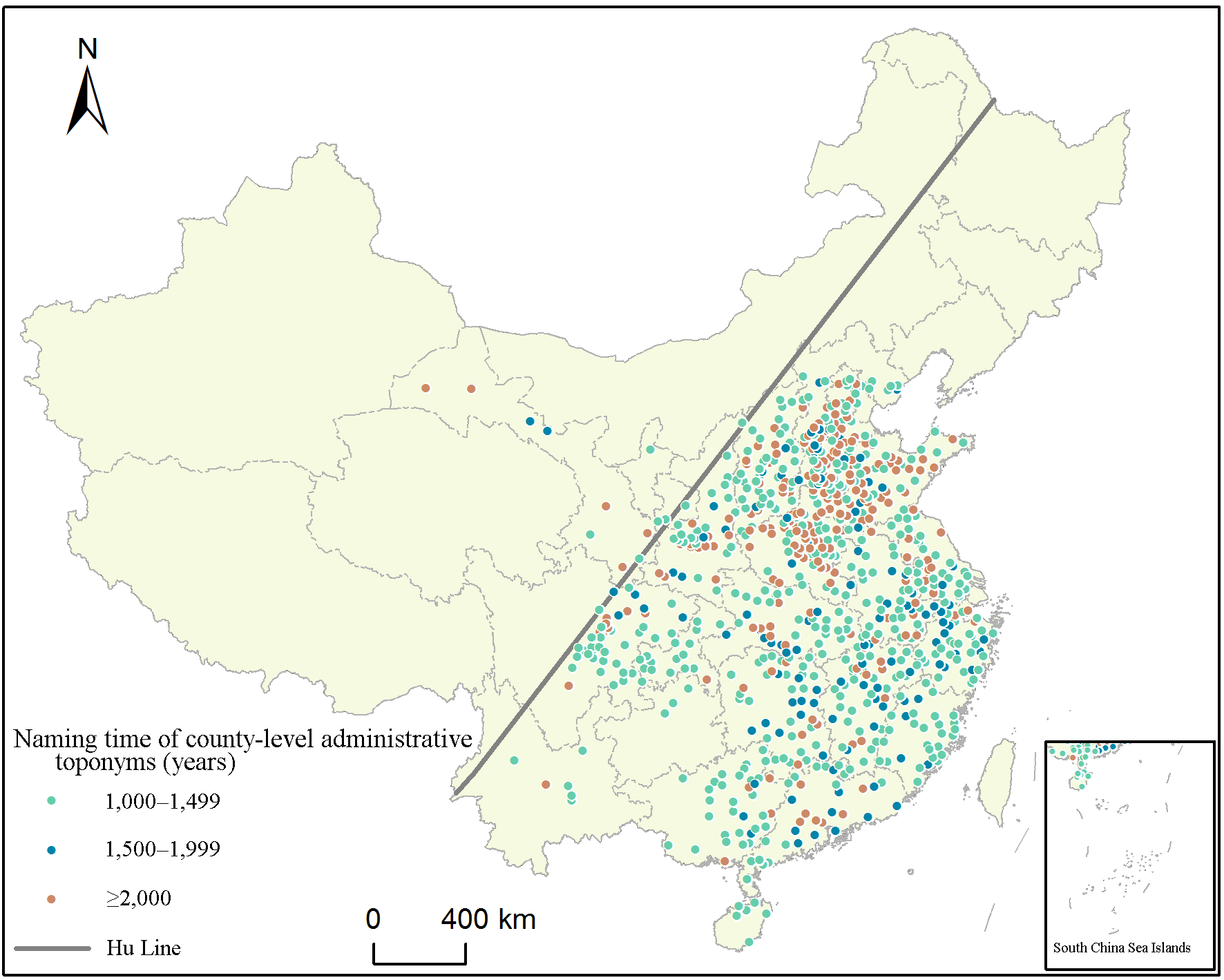

Supplement: S1 Fig — (TIF) [file pone.0217381.s001.tif]

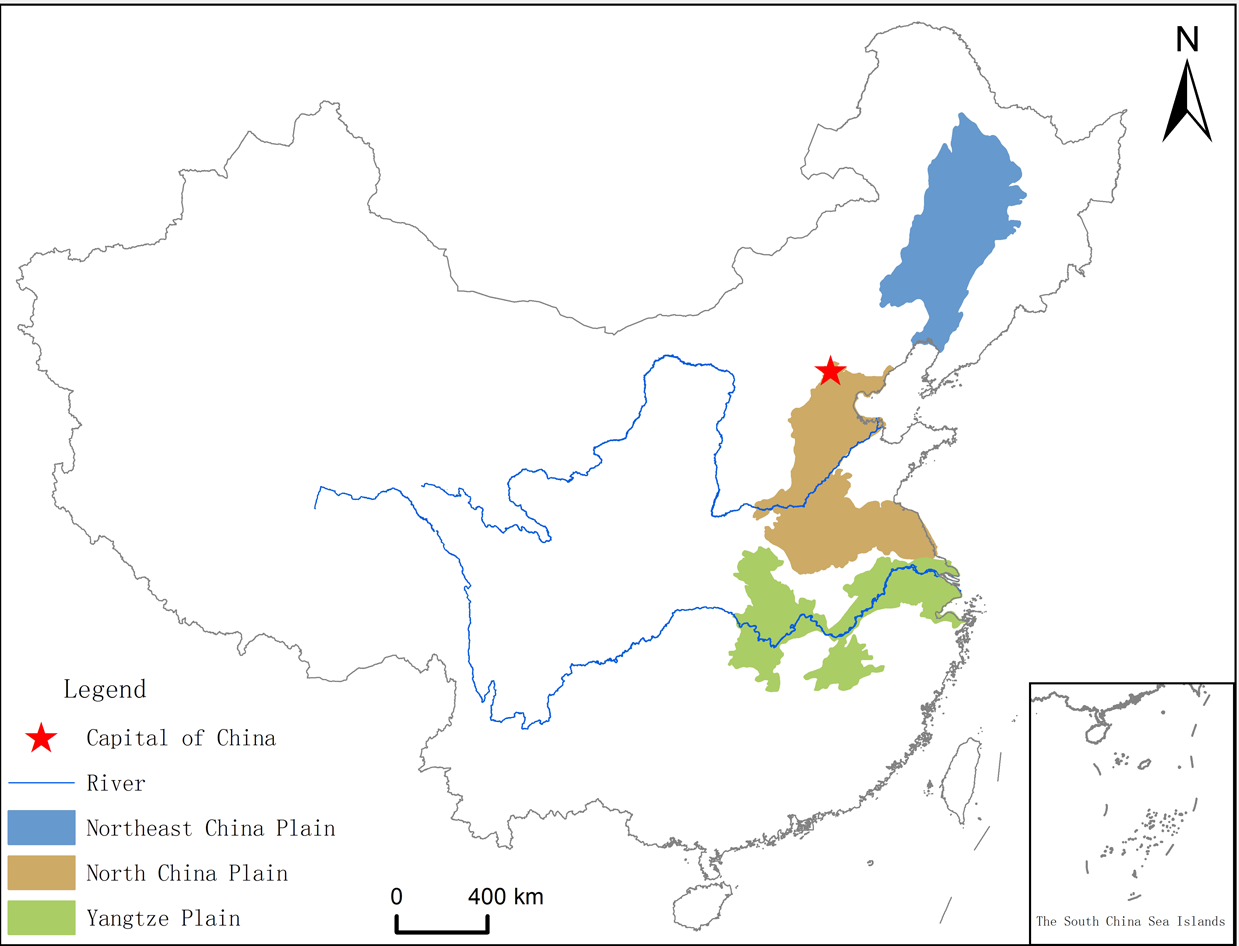

Supplement: S2 Fig — (TIF) [file pone.0217381.s002.tif]

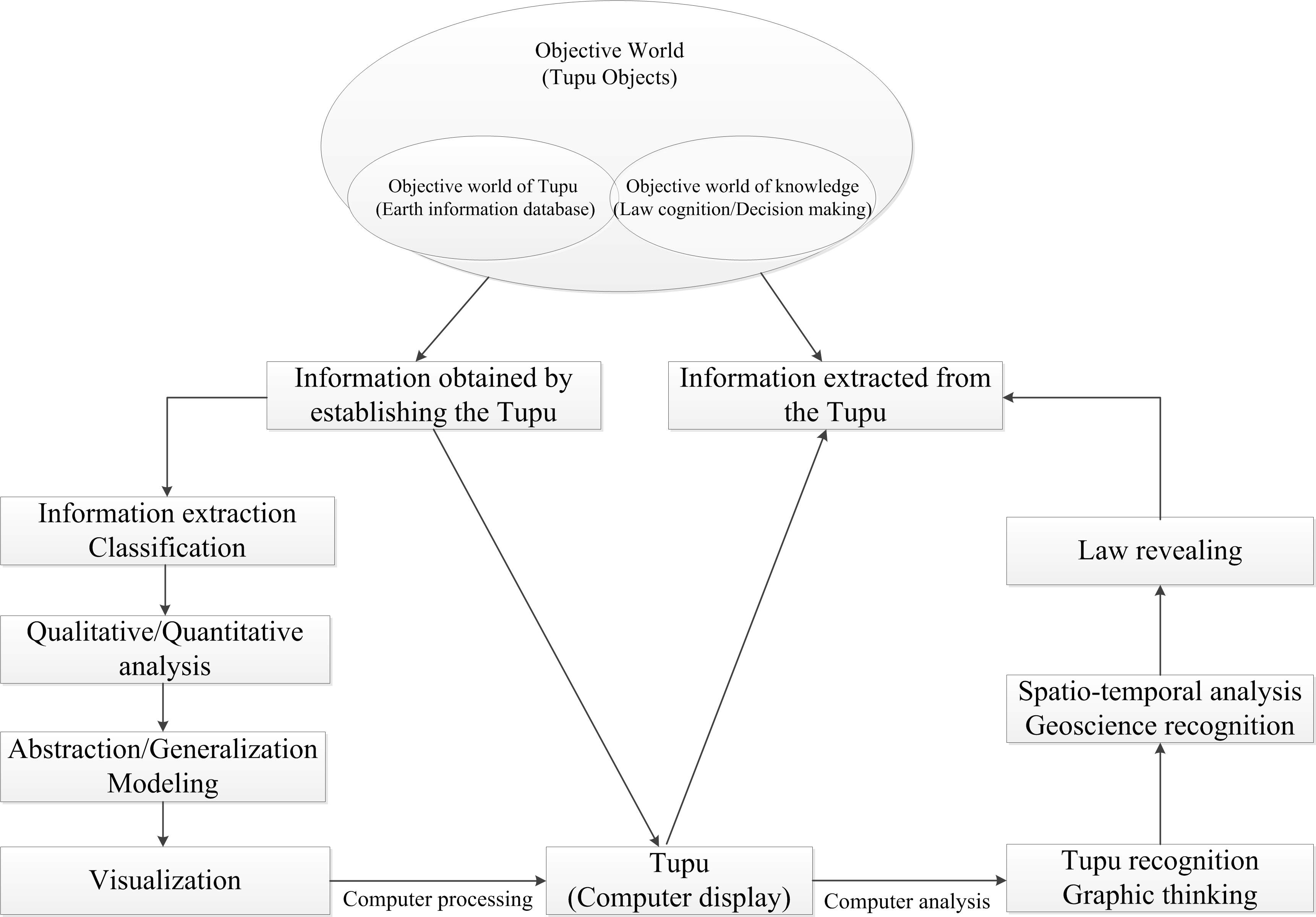

Supplement: S3 Fig — (TIF) [file pone.0217381.s003.tif]

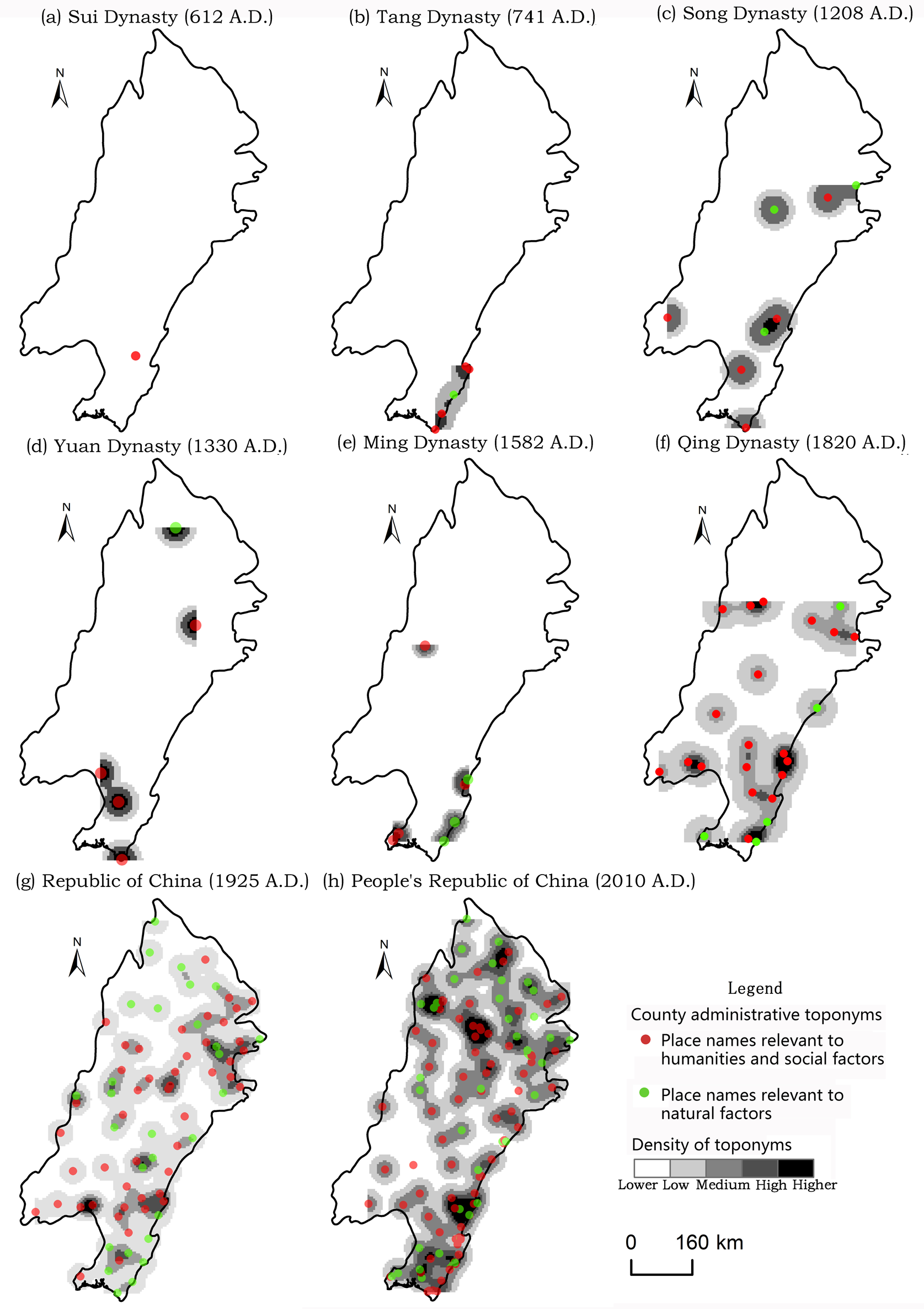

Supplement: S4 Fig — (TIF) [file pone.0217381.s004.tif]

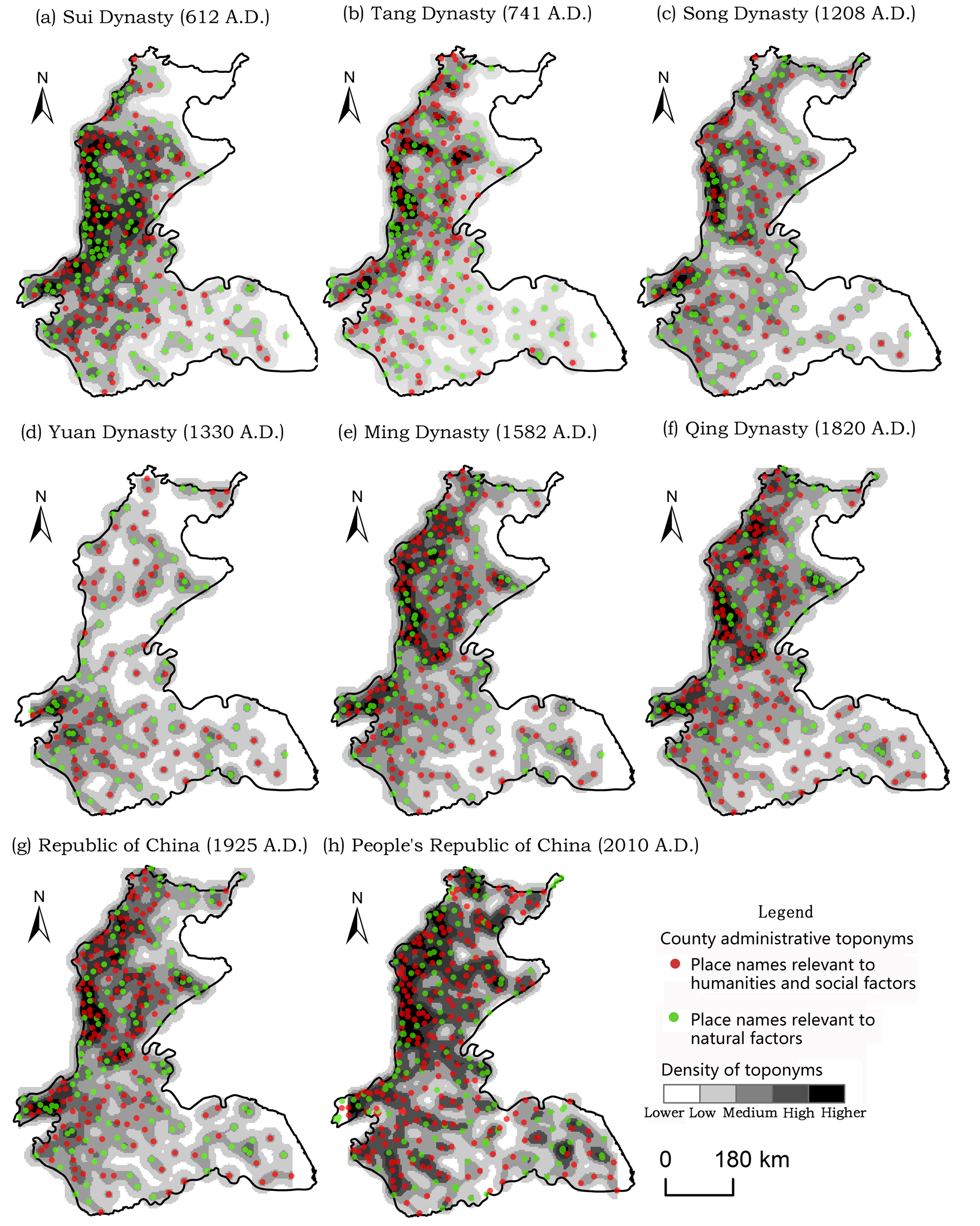

Supplement: S5 Fig — (TIF) [file pone.0217381.s005.tif]

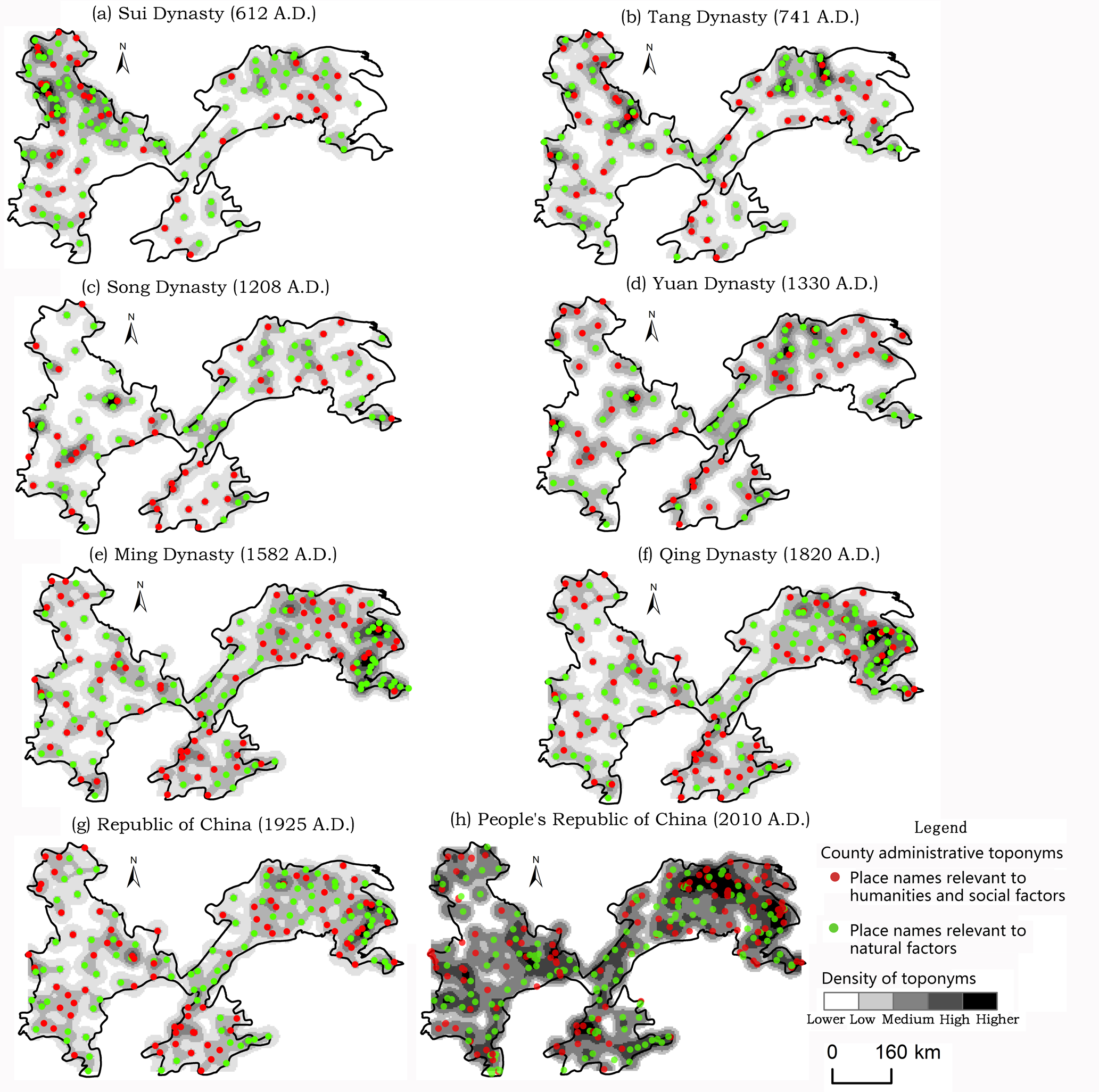

Supplement: S6 Fig — (TIF) [file pone.0217381.s006.tif]

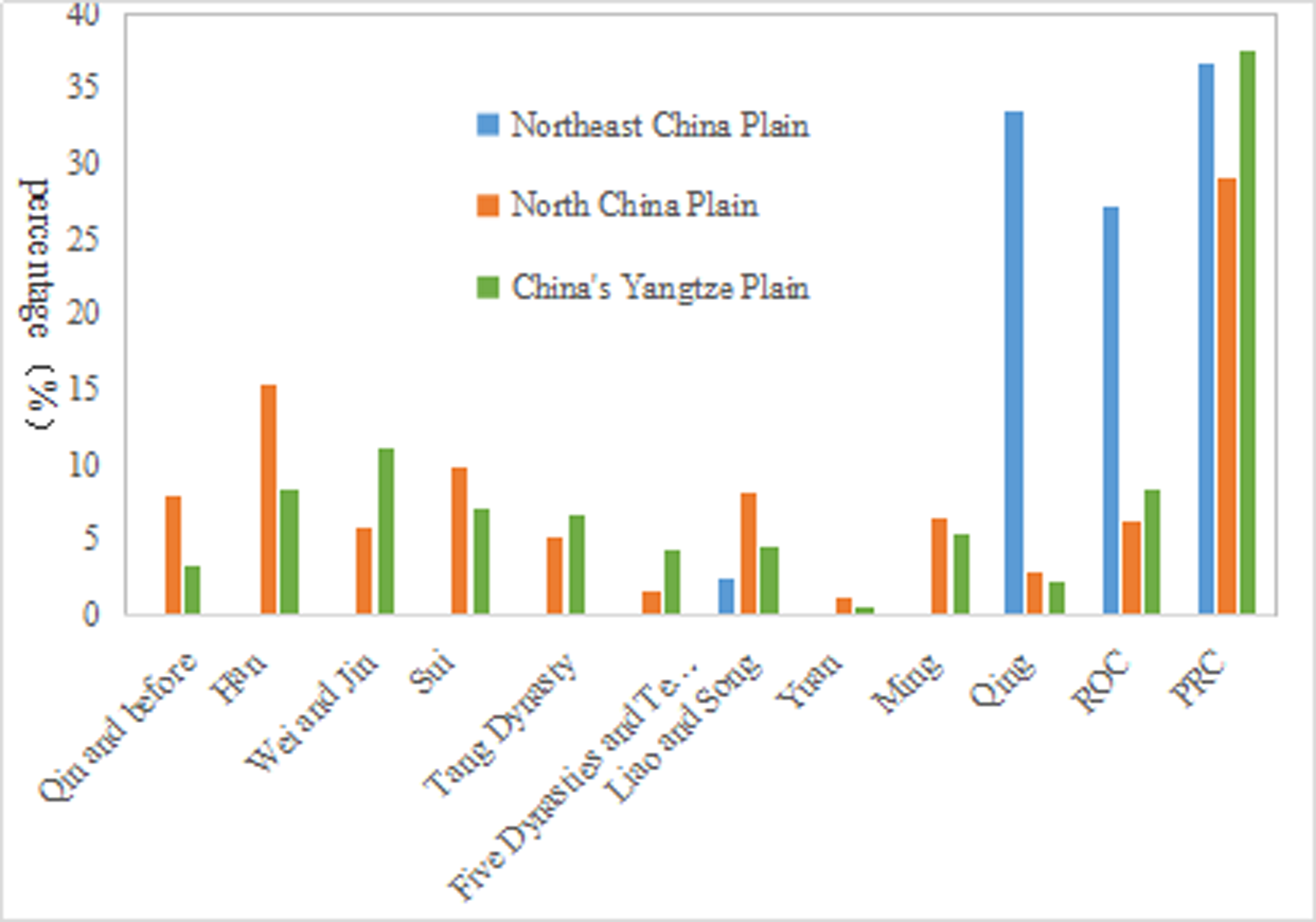

Supplement: S7 Fig — (TIF) [file pone.0217381.s007.tif]

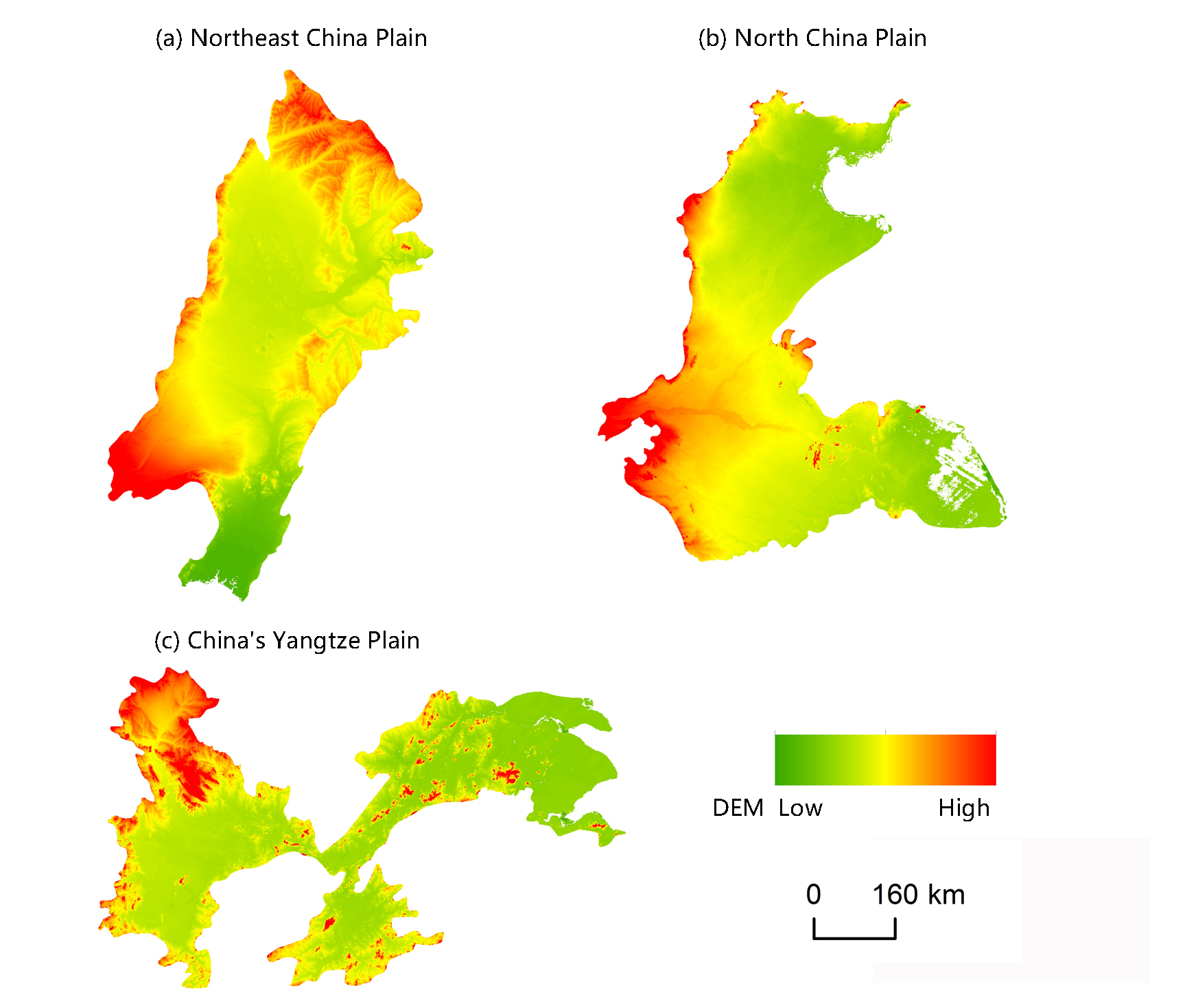

Supplement: S8 Fig — (TIF) [file pone.0217381.s008.tif]

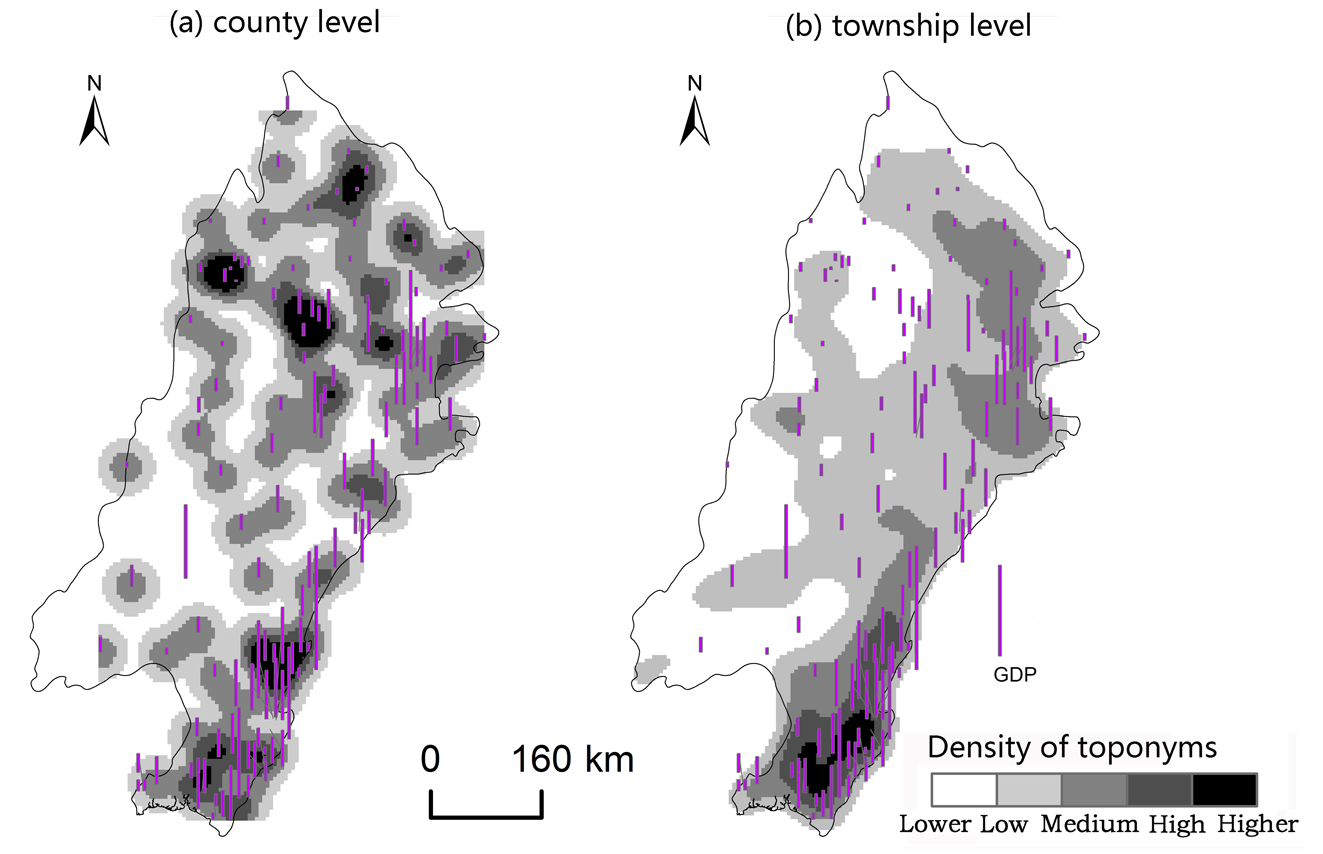

Supplement: S9 Fig — (TIF) [file pone.0217381.s009.tif]

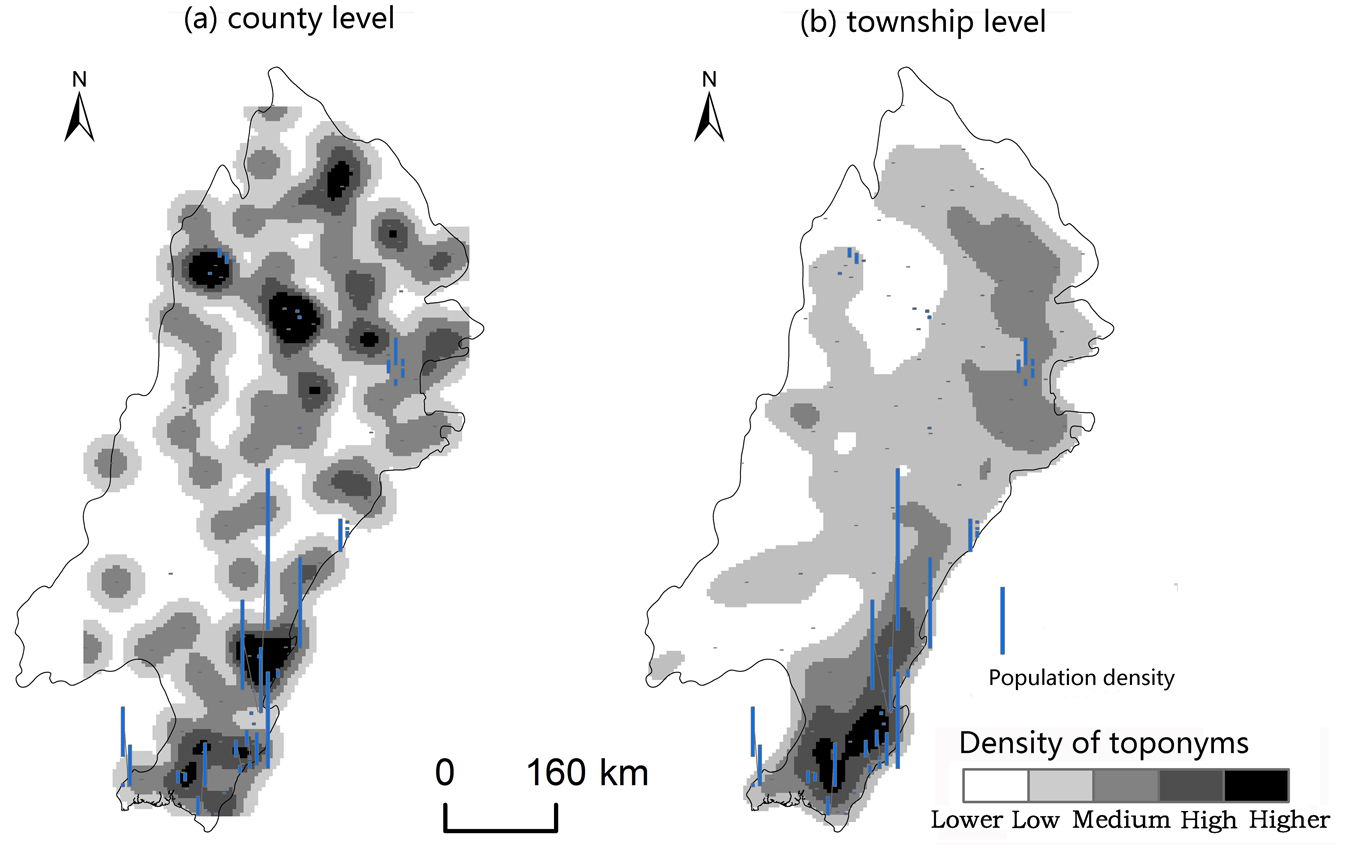

Supplement: S10 Fig — (TIF) [file pone.0217381.s010.tif]

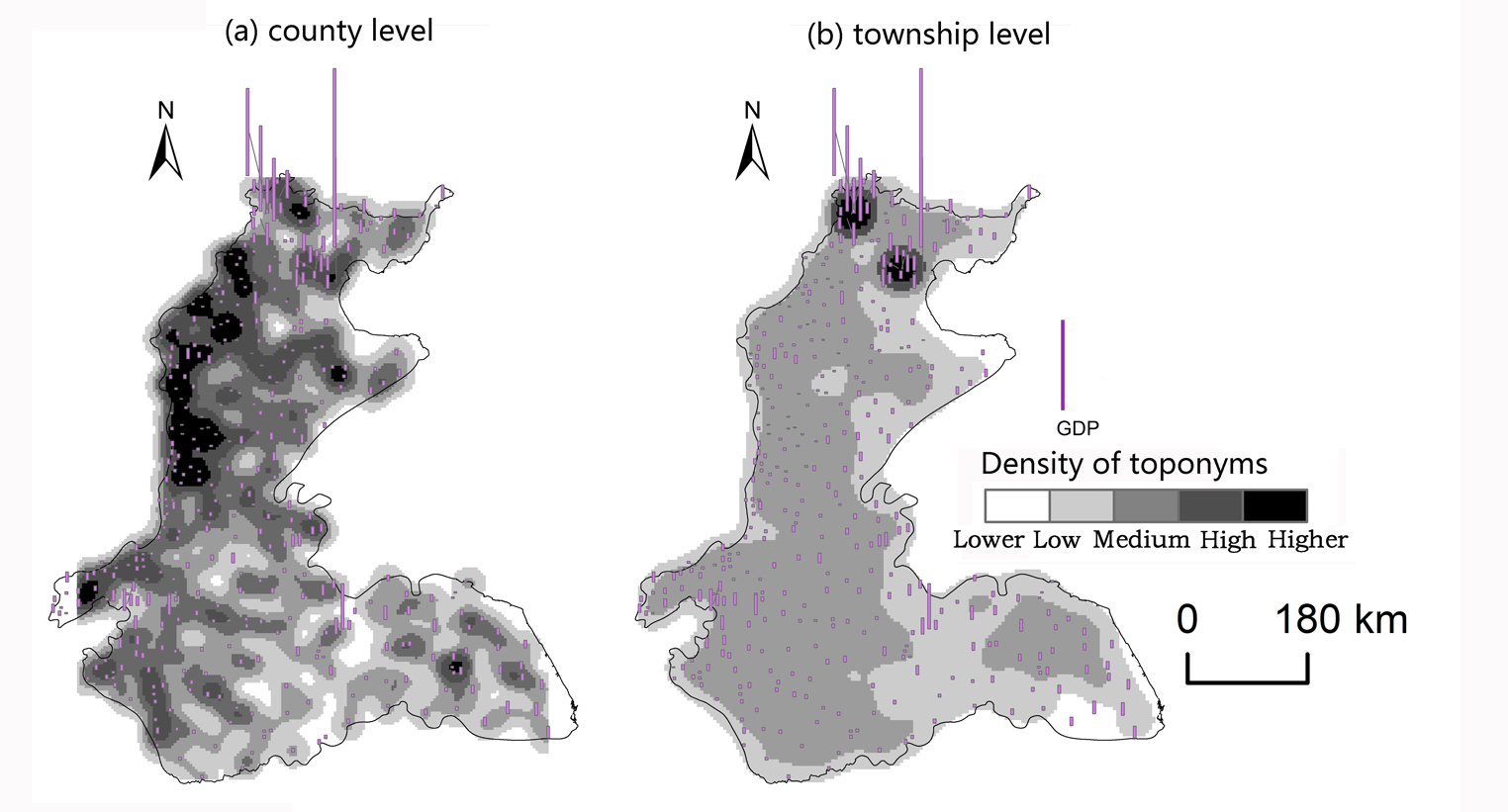

Supplement: S11 Fig — (TIF) [file pone.0217381.s011.tif]

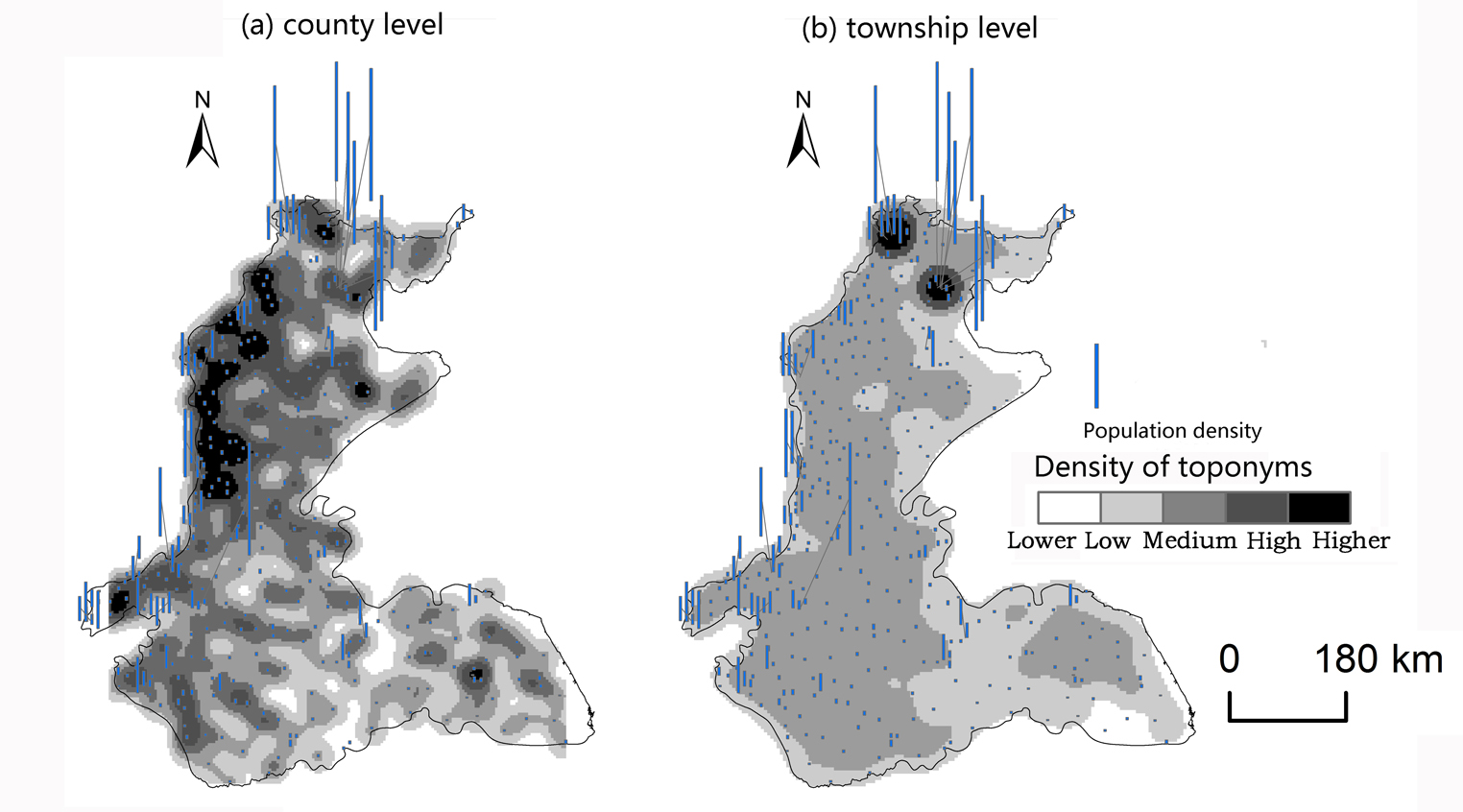

Supplement: S12 Fig — (TIF) [file pone.0217381.s012.tif]

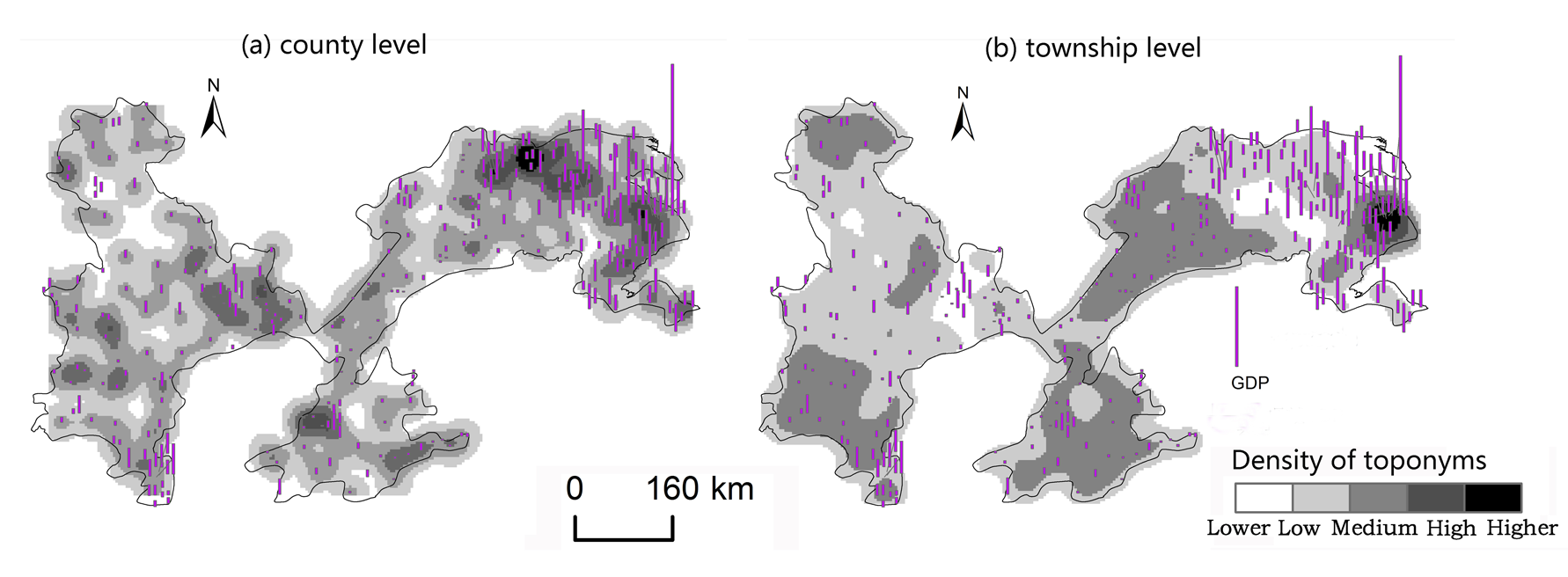

Supplement: S13 Fig — (TIF) [file pone.0217381.s013.tif]

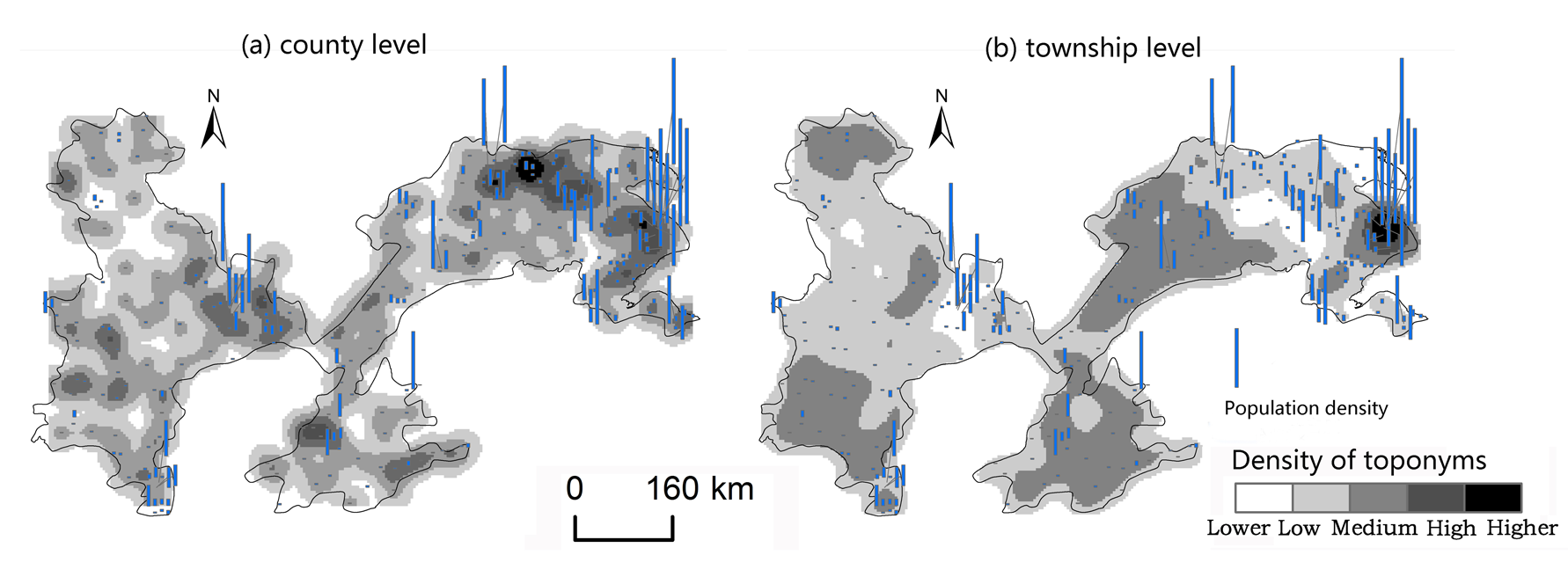

Supplement: S14 Fig — (TIF) [file pone.0217381.s014.tif]

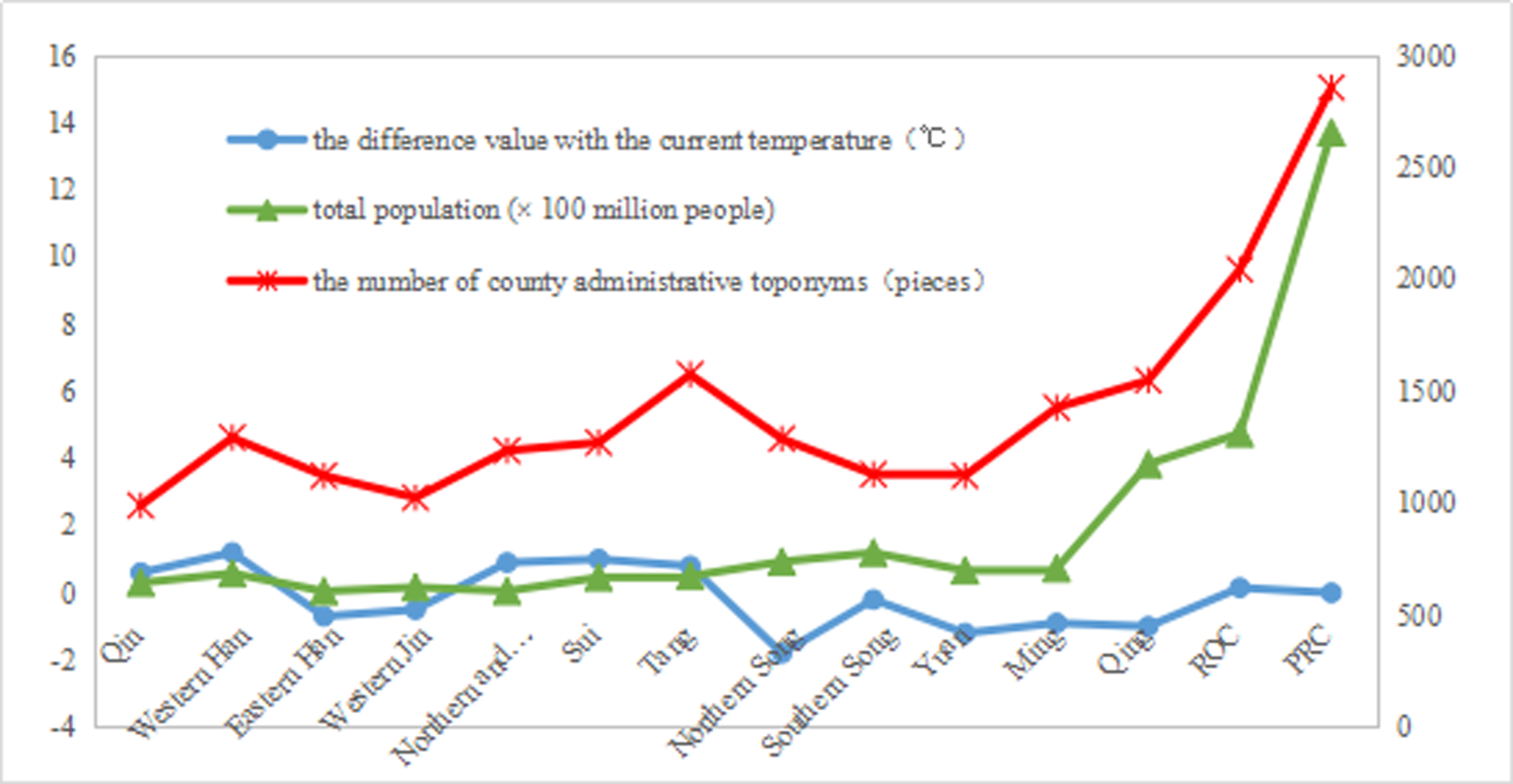

Supplement: S15 Fig — (TIF) [file pone.0217381.s015.tif]
