# Supplementary material for: Spatial-temporal characteristics and causes of changes to the county-level administrative toponyms cultural landscape in the eastern plains of China
Source: PLoS One. 2019 May 28;14(5):e0217381. doi: 10.1371/journal.pone.0217381 (PMC6538164; doi:10.1371/journal.pone.0217381)
Supplement: S1 Table — (PDF) [file pone.0217381.s016.pdf]

Table 1. Statistical characteristics of county-level administrative toponyms

Period	Number of toponyms				Average toponym density (per 10^4 km^2)			
	Northeast China Plain	North China Plain	Yangtze Plain	Whole nation	Northeast China Plain	North China Plain	Yangtze Plain	National average
Sui	1	279	132	1271	0.03	8.51	5.01	0.92
Tang	5	284	119	1575	0.16	8.67	4.51	1.13
Song	8	206	98	1129	0.26	6.29	3.72	0.81
Yuan	5	142	105	1123	0.16	4.33	3.98	0.80
Ming	7	267	178	1427	0.23	8.15	6.75	1.03
Qing	24	270	183	1549	0.79	8.24	6.94	1.11
ROC	76	280	195	2044	2.49	8.54	7.39	1.79
PRC	125	388	298	2856	4.10	11.84	11.30	2.98
