# Supplementary material for: Spatial-temporal characteristics and causes of changes to the county-level administrative toponyms cultural landscape in the eastern plains of China
Source: PLoS One. 2019 May 28;14(5):e0217381. doi: 10.1371/journal.pone.0217381 (PMC6538164; doi:10.1371/journal.pone.0217381)
Supplement: S2 Table — (PDF) [file pone.0217381.s017.pdf]

Table 2. Distribution characteristics of naming types of county-level administrative toponyms in 2010

Naming type	Northeast China Plain		North China Plain		Yangtze Plain	
	Count	(%)	Count	(%)	Count	(%)
Relevant to mountains	9	7.2	24	6.2	39	13.1
Relevant to hydrological features	30	24.0	94	24.2	102	34.2
Relevant to products	6	4.8	10	2.6	8	2.7
Relevant to terrain	4	3.2	15	3.9	6	2.0
Relevant to orientation	13	10.4	37	9.5	18	6.0
Relevant to blessings	19	15.2	70	18.0	38	12.8
Relevant to ancient relics	22	17.6	53	13.7	44	14.8
Relevant to historical figures and events	7	5.6	53	13.7	26	8.7
Relevant to surnames	3	2.4	8	2.1	5	1.7
Relevant to taboos	0	0	6	1.6	5	1.7
Other forms	12	9.6	18	4.6	7	2.3
Total	125	100	388	100	298	100
