# Supplementary material for: Spatial-temporal characteristics and causes of changes to the county-level administrative toponyms cultural landscape in the eastern plains of China
Source: PLoS One. 2019 May 28;14(5):e0217381. doi: 10.1371/journal.pone.0217381 (PMC6538164; doi:10.1371/journal.pone.0217381)
Supplement: S3 Table — (PDF) [file pone.0217381.s018.pdf]

Table 3. Statistical characteristics of county-level administrative toponyms relevant to natural factors since the Sui dynasty

Period	Northeast China Plain		North China Plain		Yangtze Plain		Total	
	Count	(%)	Count	(%)	Count	(%)	Count	(%)
Sui	0	0	151	54.1	89	67.4	240	58.3
Tang	1	20.0	140	49.3	75	63.0	216	52.9
Song	3	37.5	98	47.6	57	58.2	158	50.6
Yuan	1	20.0	65	45.8	59	56.2	125	49.6
Ming	3	42.9	116	43.4	100	56.2	219	48.5
Qing	5	20.8	111	41.1	98	53.6	214	44.9
ROC	28	36.8	109	38.9	103	52.8	240	43.6
PRC	43	34.4	141	36.3	153	51.3	337	41.6
