# Supplementary material for: Spatial-temporal characteristics and causes of changes to the county-level administrative toponyms cultural landscape in the eastern plains of China
Source: PLoS One. 2019 May 28;14(5):e0217381. doi: 10.1371/journal.pone.0217381 (PMC6538164; doi:10.1371/journal.pone.0217381)
Supplement: S4 Table — (PDF) [file pone.0217381.s019.pdf]

**Table 4. Correlation coefficients between county-level administrative toponym density and DEM, GDP, and population density**

| Plain | $R_{DEM}$ | $R_{GDP}$ | $R_{POP}$ |
|---|---|---|---|
| Northeast China Plain | -0.73 | 0.90* | 0.92* |
| North China Plain | -0.66 | 0.78* | 0.81* |
| Yangtze Plain | -0.60 | 0.88** | 0.90** |

* indicated the value was significant at the 0.05 level (2-tailed). ** indicated the value was significant at the 0.01 level (2-tailed). The grid data on China's population and GDP in 2010 (at a 1-km gridded resolution) were provided by the RESDC.
