# Supplementary material for: Spatial-temporal characteristics and causes of changes to the county-level administrative toponyms cultural landscape in the eastern plains of China
Source: PLoS One. 2019 May 28;14(5):e0217381. doi: 10.1371/journal.pone.0217381 (PMC6538164; doi:10.1371/journal.pone.0217381)
Supplement: S5 Table — (PDF) [file pone.0217381.s020.pdf]

Table 5. Correlation coefficients between administrative toponym density and DEM, GDP, and population density

Plain	County-level administrative toponym density			Township-level administrative toponym density		
	R_{DEM}	R_{GDP}	R_{POP}	R_{DEM}	R_{GDP}	R_{POP}
Northeast China Plain	-0.68	0.75*	0.89**	-0.78	0.80*	0.85*
North China Plain	-0.62	0.81*	0.83*	-0.59	0.89*	0.91*
Yangtze Plain	-0.56	0.76**	0.85**	-0.54	0.93**	0.95**

* indicated the value was significant at the 0.05 level (2-tailed). ** indicated the value was significant at the 0.01 level (2-tailed). The GDP data in 2010 at the county level were from the RESDC and population data at the county level from the 6th Census of China (2010).
